# High yield engineered nanovesicles from ADSC with enriched miR-21-5p promote angiogenesis in adipose tissue regeneration

**DOI:** 10.1186/s40824-022-00325-y

**Published:** 2022-12-17

**Authors:** Di Sun, Shan Mou, Lifeng Chen, Jie Yang, Rongrong Wang, Aimei Zhong, Wei Wang, Jing Tong, Zhenxing Wang, Jiaming Sun

**Affiliations:** 1 Department of Plastic Surgery, Union Hospital, Tongji Medical College, Huazhong University of Science and Technology, Wuhan, 430022 China; 2 Wuhan Clinical Research Center for Superficial Organ Reconstruction, Wuhan, 430022 China

**Keywords:** Engineered nanovesicles, MSCs, Angiogenesis, Adipose tissue regeneration, miRNAs

## Abstract

**Background:**

Mesenchymal stem cell-derived extracellular vesicles (MSC-EVs) have been found to have a great potential for soft tissue repair due to various biological functions, including pro-angiogenesis and low immunogenicity. However, the low yield and heterogeneity of MSC-EVs limited their clinical transformation. This study was designed to develop a novel adipose-derived stem cell engineered nanovesicles (ADSC-NVs) with high production and explore its pro-angiogenetic effect and application in adipose tissue regeneration.

**Methods:**

Adipose-derived stem cell-derived extracellular vesicles (ADSC-EVs) were isolated from an EVs-free culture medium for human ADSCs (hADSCs). ADSC-NVs were prepared by sequentially extruding ADSCs followed by iodixanol density gradient ultracentrifugation and were compared with ADSC-EVs in morphology, size distribution, protein contents and yield. The pro-angiogenetic effect of ADSC-NVs in different doses (0, 5, 20 and 80 μg/mL) in vitro was determined using transwell assay, tube formation assay, western blot and qRT-PCR. In vivo, BALB/c nude mice were administered injection of a mixture of fat granules and different dose of ADSC-NVs and grafts were harvested at 12 weeks post-transplantation for further analysis. By analyzing the weight and volume of grafts and histological evaluation, we investigated the effect of ADSC-NVs in vessel formation and adipose tissue regeneration.

**Results:**

Our results showed yield of purified ADSC-NVs was approximately 20 times more than that of ADSC-EVs secreted by the same number of ADSCs. In vitro, both ADSC-NVs and ADSC-EVs exhibited a dose-dependent pro-angiogenetic effect, despite their distinct miRNA profiles. These effects of ADSC-NVs may be mediated by enriched miR-21-5p via PTEN inhibition and PI3K/p-Akt signaling activation. Furthermore, after a mixed injection of ADSC-NVs, vessel formation and adipose regeneration were observed in vivo *in* fat implants.

**Conclusions:**

Our study developed a potent alternative of ADSC-EVs. ADSC-NVs have a high pro-angiogenesis potential and can be used as cell-free therapeutic biomaterials in soft tissue regeneration.

**Supplementary Information:**

The online version contains supplementary material available at 10.1186/s40824-022-00325-y.

## Background

Mesenchymal stem cells (MSCs) play a critical role in angiogenesis and tissue regeneration due to their ability to differentiate into vascular and vascular support cells [1] and their paracrine role through factors [2, 3]. Mesenchymal stem cell-derived extracellular vesicles (MSC-EVs) are important paracrine communication soluble mediators in both physiological and pathological conditions. MSC-EVs are membrane-packed vesicles secreted by MSCs with a diameter of 50–200 nm. They contain numerous miRNAs as well as other biomolecules such as proteins, lipids, mRNA, and miRNA [4]. Transfer of these contents can mediate intercellular interactions and modulate several processes, including pro-angiogenesis [2, 5], anti-inflammation [2, 6], and anti-apoptosis [7, 8], all of which can promote tissue regeneration [9, 10]. Therefore, MSC-EVs have cell-free therapeutic potential for regenerative medicine applications such as bone reconstruction, traumatic myocardium repair, and adipose tissue regeneration.

Currently, fat grafting is an effective strategy for reconstructing congenital and acquired soft tissue defects caused by sequelae of mastectomy, radiation therapy, trauma, and facial rejuvenation. Notably, the survival rate of transplanted fat tissue ranges from 20 to 80%, which is mainly due to delayed and insufficient re-vascularization [2, 11]. Angiogenesis is the process by which new vessels form. The PI3K/Akt pathway is strongly linked to angiogenesis regulation because it is required for both angiogenesis-induction and vascular permeability maintenance [12]. Moreover, activating the angiogenic switch necessitates the introduction of pro-angiogenic factors, among which MSC-EVs are capable of supporting endothelial cell viability and proliferation [13]. The miRNAs found in extracellular vesicles could act as an angiogenic switch, mediate downstream signaling pathways, modulate the angiogenic process, and ultimately promote tissue repair [14].

Despite the promising future of MSC-EVs in regenerative medicine, several limitations restrict their application in large-scale production. Notably, MSC expansion is essential for MSC-EVs preparation, which may result in cell senescence, variable differentiation potentials, and other passage-specific transformations, resulting in MSC-EVs heterogeneity [15]. MSCs secret extracellular vesicles in extremely small amounts, and slight change in EVs preparation could lead to great difference in the final production yield. In terms of protein quantification, ultracentrifugation-based isolation of EVs from cell culture conditioned media could yield around 1.6 ~ 10 μg/mL EVs per day [16–19]. In terms of particle quantification, MSCs from bone marrow and adipose tissue release ~ 200 EVs per cell in 24 h, whereas umbilical cord mesenchymal stem cells could yield 4 times as many EVs per cell than that of ADSCs [16]. However, to achieve therapeutic outcomes, typically a dose of 10^9^ ~ 10^11^ EVs is needed in the treatment in mice as reported [20], which may require time-consuming conditioned media collection and cell expansion and thus limit its large-scale application [19]. Furthermore, the time-consuming and complicated purification process severely limits the amount of MSC-EVs that may be sufficiently purified for clinical use [21].

Several studies have looked into ways to increase the yield of MSC-EVs. The major strategies include optimizing MSC culture methods and modification of MSC-EVs isolation methods. Approaches for optimizing MSC expansion have been developed, including the use of small-molecule drugs as MSC proliferation modulators [22], three-dimensional (3D) culturing systems for large-scale MSC amplification [23], and microtissues to mimic an in vivo environment [24]. Extracellular vesicles secreted by dermal fibroblast spheroids cultured in suspension exhibit a significant increase in levels of proteins involved in collagen fiber maintenance and hence are used to prevent skin aging [25]. Besides, stimulations such as hypoxia condition [26], and chemical treatment [27] could activate the metabolic pathway, DNA damage, and other processes, and thus augment EVs shedding. Top-down bioengineering of nanovesicles (NVs) was one of the successful strategies for modifying MSC-EVs isolation methods. Two major strategies used include: 1) sequential extrusion of cells through micro-filters based on liposome preparation technology [28–32]; and 2) use of specific microfluidic devices [33, 34], in which cells are subjected to shear stress and cut into bilayer membrane. In summary, parent cells are crushed and fragmented into lipid bilayer fragments. The fragments spontaneously self-assemble to form spheres because of the amphiphilic properties of lipids in an aqueous solution [29]. NVs derived from embryonic stem (ES) cells were first developed in 2014 [28], providing a new strategy to overcome the aforementioned yield efficiency limitations of EVs. Biochemical profiling revealed that these ESC-NVs morphologically resembled naturally secreted extracellular vesicles and improved proliferation in primary murine skin fibroblasts in a manner similar to ESC-EVs.

**Fig. 1:**
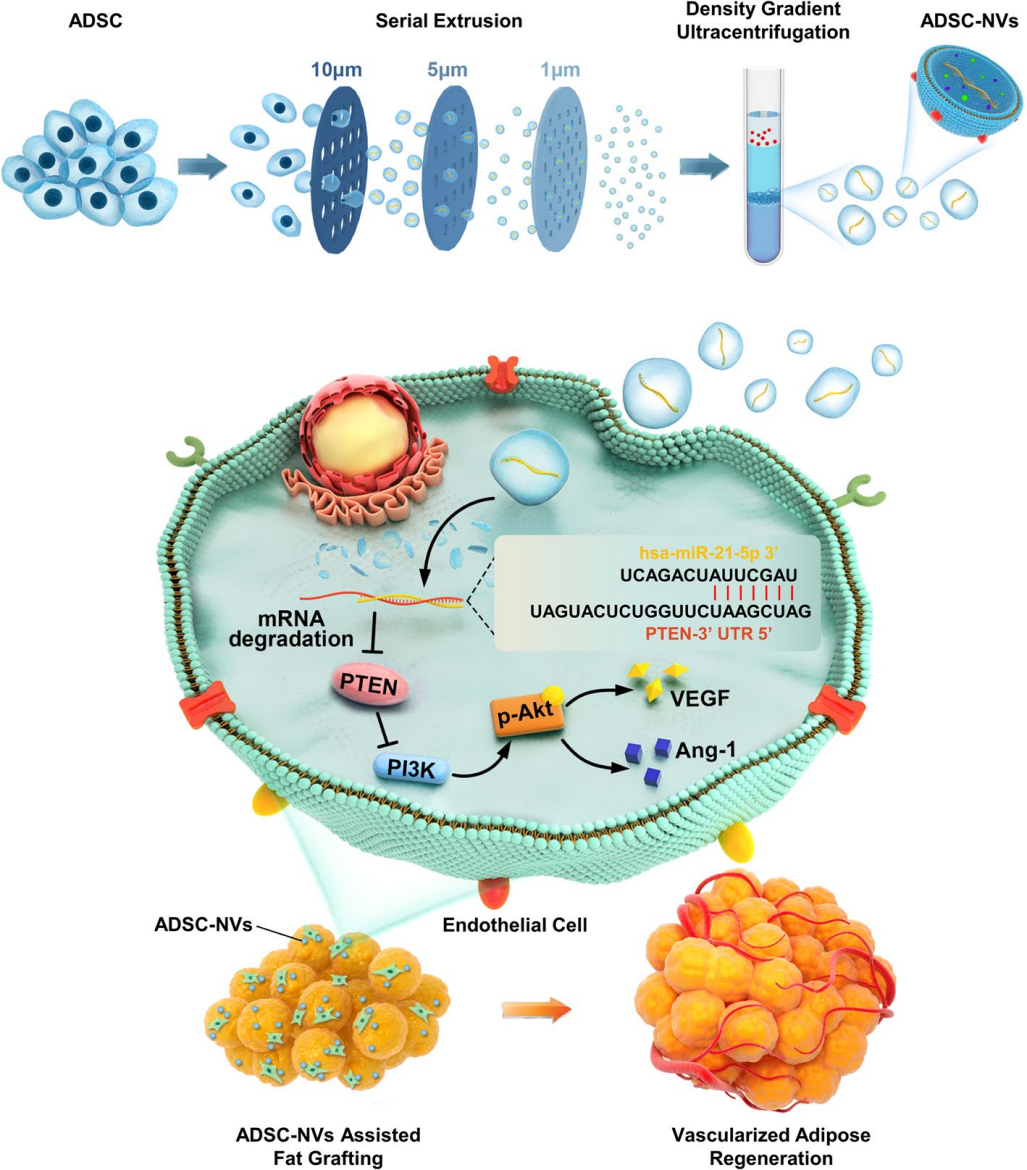
Schematic diagram showing preparation of adipose derived stem cell-engineered nanovesicles (ADSC-NVs) for development of angiogenesis through modulating the PTEN/PI3K/Akt signaling pathway and promotion of vascularized adipose regeneration

To improve on the current MSC-EVs-based tissue regeneration strategy, adipose-derived stem cell-engineered nanovesicles (ADSC-NVs) were developed to mimic extracellular vesicles in scalable production yields with similar angiogenic capabilities (Fig. 1). First, human ADSCs (hADSCs) were sequentially extruded through micro-filters to engineer NVs from ADSCs, resulting in a high yield of extracellular vesicles. Second, the in vitro pro-angiogenesis effect of ADSC-NVs and ADSC-EVs was investigated. Third, the microRNA expression profile of ADSC-NVs and ADSC-EVs was analyzed by miRNA sequencing. Fourth, in the fat transplantation model, implantation of ADSC-NVs promoted regeneration of vascularized adipose tissue. A high level of miR-21-5p was detected in ADSC-NVs, which promoted angiogenesis by inhibiting PTEN and activating PI3K/Akt signaling. These findings suggest that ADSC-NVs are a promising cell-free therapeutic material for graft vascularization, adipose tissue engineering, regenerative medicine, and other soft tissue engineering.

## Methods

### Preparation of ADSCs

This study was approved by the Ethics Committee of the Huazhong University of Science and Technology. ADSCs isolated from healthy female donors via lipo-aspiration were obtained from Wuhan Union Hospital. Tissue samples were washed three times with phosphate-buffered saline (PBS) and digested using 0.2% NB4 collagenase (SERVA, 17454) at 37 °C for 2 h. After centrifugation at 280 g for 5 min, cells from pellet were maintained in DMEM (Hyclone) medium supplemented with 10% fetal bovine serum (FBS; Hyclone, SV30208.02) and 1% penicillin-streptomycin antibiotic at 37 °C in a humidified atmosphere containing 5% CO_2_. Notably, cells at passages 3–6 were used in subsequent analyses.

### Isolation of ADSC-derived extracellular vesicles and engineered nanovesicles

Extracellular vesicles (EVs) were extracted from ADSCs culture media through density gradient ultracentrifugation as previously described [19]. Briefly, ADSCs were cultured in a medium containing 10% EV-free FBS (Hyclone). FBS was depleted of bovine exosomes by ultracentrifugation at 100,000 g for 70 min. After culturing for 48 h, cells and debris were removed from the supernatant by centrifugation at 300 g for 10 min at 4 °C. The supernatant was then sequentially centrifuged at 2000 g for 10 min and 10,000 g for 30 min. EVs were then purified by ultracentrifugation (Thermo Fisher Scientific, Sorvall WX 80+) at 100,000 g for 70 min and resuspended in PBS.

ADSCs from passage 3 to 6 were used for preparation of NVs. Cells at > 90% confluence were detached using cell scraper and resuspended in 1 mL PBS, followed by sequential extrusion of the suspension five times through filter membranes (Whatman) with decreasing apertures (10 μm, 5 *μ*m, and 1 *μ*m) using a mini-extruder (Avanti Polar Lipids). Finally, NVs were separated using bottom-up iodixanol density gradient ultracentrifugation. Notably, Opti-prep (AXIS-SHIELD) working solution was prepared from a discontinuous iodixanol gradient (30–10% Opti-prep solution) in an ultracentrifuge tube. After extrusion, the solution was loaded on top of the Opti-prep working solution and the tube was ultra-centrifuged at 10,000 g for 70 min. The final NVs pellet was resuspended in PBS.

### Characterization and analysis of ADSC-EVs and ADSC-NVs

A transmission electron microscope (TEM, HITACHI, HT7800) was used to determine purity of the ADSC-EVs and ADSC-NVs. The purified ADSC-EVs and ADSC-NVs were placed into formvar/carbon-coated grid and rinsed after absorption for 30 min. The TEM grid was negatively stained with 2% uranyl acetate and examined after drying. Size and particle concentration of ADSC-EVs and ADSC-NVs were determined using Nanoparticle Tracking Analysis (NanoSight, Malvern Instruments) equipped with a blue laser (405 nm), whereas the protein concentration was determined through BCA protein assay (Beyotime Biotechnology, P0012) following the manufacturer’s instructions.

### Cellular uptake assay

To explore the intracellular internalization, ADSC-EVs and ADSC-NVs were labeled with PKH26 red fluorescent cell linker (Sigma, MINI26-1KT) according to the manufacturer’s instructions. Human umbilical vein endothelial cells (HUVECs) purchased from the American Type Culture Collection (Rockville, Md.) were used as recipient cells. The cells were incubated in endothelial cell medium (ScienceCell, #1001) supplemented with 5% fetal bovine serum (FBS; ScienceCell, #0025), 1% endothelial cell growth supplement (ECGS; ScienceCell, #1052) and 1% penicillin-streptomycin antibiotic. HUVECs were incubated with PKH26-labeled EVs or NVs in equivalent number of particles for 1, 2, 3 or 4 h, washed three times with PBS, and then stained using Actin-Tracker Green-488 (Beyotime Biotechnology, C2201S). The nuclei were stained using DAPI staining solution (Beyotime Biotechnology, C1006). Samples were observed under a confocal scanning microscope (Nikon, C2+). The internalization rate of ADSC-EVs and ADSC-NVs was evaluated by calculating area ratio between ADSC-EVs/ ADSC-NVs and HUVECs using ImageJ software.

### Transwell assay

Considering the biological effect and their further practical application, different doses of ADSC-NVs and ADSC-EVs (ranging from 5 to 80 μg/mL) were administered to the same initial number of HUVECs to evaluate the angiogenic potency of ADSC-NVs and ADSC-EVs. Transwell assays were performed using polycarbonate membrane Transwell inserts with a pore size of 8 μm (BIOFIL, TCS-003-024) to perform determine the migratory capacity of treated and untreated HUVECs. Briefly, 5 × 10^4^ HUVECs suspended in serum-free RPMI medium were seeded into the upper chamber, whereas 650 μL RPMI medium containing 0, 5, 20, and 80 μg/mL ADSC-NVs or ADSC-EVs was added to the lower chamber. After incubation for 24 h, the upper chamber was washed three times with PBS and then fixed with 4% paraformaldehyde for 30 min. Non-migrating cells over the membrane were carefully wiped off with a cotton swab from the inside of the upper chamber. The membrane was dried and the cells on the bottom were stained with crystal violet staining solution (Beyotime Biotechnology, C0121) for 15 min. Stained cells were visualized under a standard bright field microscope, and a total of 5–6 microscope fields were randomly selected to determine cells that had migrated.

### Tube formation assay

First, 50 μL growth factor reduced matrigel (Corning, 356,230) was added to each well of a 96-well plate on ice and the plate was placed at 37 °C for 30 min. Next, HUVEC-GFP were resuspended with FBS-free RPMI containing different concentrations (0, 5, 20, and 80 μg/mL) of ADSC-NVs or ADSC-EVs, followed by seeding the cells into each well at a concentration of 2 × 10^4^ cells/well. After incubation for 6 h, tube formation was photographed using a confocal scanning microscope (Nikon, C2+). Branches of blood vessels were measured to examine the total tube length using ImageJ software.

### Cell apoptosis assay

The Annexin V-FITC/PI apoptosis detection kit (Elabscience, E-CK-A211) was used to determine the effect of ADSC-EVs and ADSC-NVs on apoptosis of HUVECs according to the manufacturer’s instructions. Briefly, HUVECs were incubated with different concentrations of ADSC-EVs or ADSC-NVs (0, 5, 20, and 80 μg/mL) for 24 h, and then they were collected and resuspended in 500 μL binding buffer. Next, cells were incubated with 5 *μ*L of Annexin V-FITC and 5 *μ*L of propidium iodide (PI) for 10 min in the dark, followed by flow cytometry analysis. Notably, Annexin V+/PI- was defined as early apoptotic stage, Annexin V+/PI+ was defined as apoptotic cells, and Annexin V−/PI+ was defined as dead cells. Total apoptotic rate was the sum of early apoptotic rate and apoptotic rate.

### RNA extraction and miRNA-seq

Total RNA was extracted from ADSC-NVs and ADSC-EVs using SeraMir RNA purification kit (System Biosciences, RA808A-1) in accordance with the manufacturer’s protocol. RNA quality was examined using Nanodrop™ One C spectrophotometer, and qualified RNAs were subsequently quantified using Qubit3.0 with Qubit™ RNA Broad Range Assay kit (Life Technologies, Q10210). miRNA sequencing library was obtained using Small RNA Sample Pre Kit (TruSeq, Illumina) following the manufacturer’s instructions. Small RNAs were reverse transcribed and amplified through PCR, followed by sequencing of PCR products using Illumina NovaSeq 6000 platform. For miRNA-seq data analysis, raw reads were filtered using fastx_toolkit (version: 0.0.13.2) and adaptor sequences were removed using cutadapt (version: 1.15). The target genes of miRNA in ADSC-NVs and ADSC-EVs were predicted using miRanda (http://www.microrna.org/). Finally, gene ontology (GO) and Kyoto Encyclopedia of Genes and Genomes (KEGG) enrichment analyses of the predicted target genes were performed using DAVID Bioinformatics.

### Quantitative reverse transcription-polymerase chain reaction (qRT-PCR) analysis

All primers used for mRNA detection were synthesized by Wuhan Biofavor Biotech, whereas all primers used for miRNA detection were synthesized by Biossci Biotechnology. Table 1 shows sequences of all primers used for qRT-PCR. qRT-PCR was performed using AceQ Universal SYBR qPCR Master Mix (Vazyme) following the manufacturer’s protocol. The reaction volumes contained 5 μL diluted cDNA solution, 10 *μ*L Mix, and 0.4 *μ*L of forward and 0.4 *μ*L of reverse primers. qRT-PCR was performed using the StepOnePlus™ Real-Time PCR System (Applied Biosystems) with the following thermocycling conditions: denaturation at 95 °C for 5 min, followed by 40 cycles of 95 °C for 10 s and 60 °C for 30 s. Relative miRNA expression was normalized to U6 miRNA and calculated using the 2^−ΔΔCT^ method.

**Table 1:** Primers used for qRT-PCR analysis

Gene name	Forward sequence (5′-3′)	Reverse sequence (5′-3′)
U6	CTCGCTTCGGCAGCACATA	CGAATTTGCGTGTCATCCT
hsa-miR-21-5p	GCCGCGTAGCTTATCAGACT	CAGTGCAGGGTCCGAGGTAT
hsa-miR-199a-3p	CGCCGACAGTAGTCTGCACAT	CAGTGCAGGGTCCGAGGTAT
hsa-miR-16-5p	GCCGTAGCAGCACGTAAATA	CAGTGCAGGGTCCGAGGTAT
hsa-let-7a-5p	CGCCGTGAGGTAGTAGGTTGT	CAGTGCAGGGTCCGAGGTAT
hsa-miR-26a-5p	CCGCCGTTCAAGTAATCCAGG	CAGTGCAGGGTCCGAGGTAT
Mus PTEN	GAAAGGGACGGACTGGTGTA	AGTGCCACGGGTCTGTAATC
Mus PI3K	ACCTGGACTTAGAGTGTGCC	TCAGCAGTGTCTCGGAGTTT
Homo ANG1	GGACAGCAGGAAAACAGAGC	GCCCTTTGAAGTAGTGCCAC
Homo VEGF	GGAGGAGGGCAGAATCATCA	CTTGGTGAGGTTTGATCCGC
Homo Bax	AAGAAGCTGAGCGAGTGTCT	GTTCTGATCAGTTCCGGCAC
Homo Bcl-2	GCCTTCTTTGAGTTCGGTGG	GAAATCAAACAGAGGCCGCA
Homo b-actin	CCCTGGAGAAGAGCTACGAG	CGTACAGGTCTTTGCGGATG

### Dual-luciferase reporter system

We prepared luciferase plasmids containing wild-type (pYr-MirTarget-homo PTEN 3’UTR) or mutated (pYr-MirTarget-homo PTEN 3’UTR-mut) putative PTEN binding sites for miR-21-5p targeting. HEK-293 cells were first plated in a 6-well plate at 5 × 10^5^ cells/well and transfection was only performed once the cells achieved 60–70% confluence. Two hours before transfection, the complete medium was replaced with serum-free medium. Lipofectamine 2000 (Invitrogen Life Technologies, 780,373) was used to transfect the cells. Each plasmid construct was co-transfected with miR-21-5p mimics or a corresponding negative control (NC). Cells were lysed 24 h after transfection, and miRNA expression was determined using Dual-Luciferase Reporter Assay System (Beyotime Biotechnology, RG027) following the manufacturer’s instructions.

### Western blot assay

ADSC-EVs, ADSC-NVs, cells, or tissues were lysed using RIPA buffer (Beyotime Biotechnology, P0013B) containing 1% PMSF (Aladdin, P105539), and the supernatant from lysates was used for subsequent western blot analysis. Protein samples were resolved by 10% SDS-PAGE and then transferred onto a PVDF membrane (Millipore, IPVH00010). After blocking in TBST with 5% non-fat milk for 2 h, membranes were incubated at 4 °C overnight with the following primary antibodies: CD29 (Novus, NBP2–36561), TSG101 (Servicebio, GB11618), ANG1 (Proteintech, 23,302–1-AP), VEGF (Boster Bio, PB0084), PTEN (Proteintech, 22,034–1-AP), PI3K (Proteintech, 60,225–1-AP), Akt (Affinity, AF6261), and p-Akt (Affinity, AF0016). On the next day, membranes were washed with TBST and hybridized with horseradish peroxidase (HRP)-conjugated goat anti-rabbit (Boster Biological Technology, BA1054) and anti-mouse (Boster Biological Technology, BA1051) IgG antibodies for 2 h at room temperature. Finally, bands were visualized using ECL blotting detection reagents (Servicebio, G2014).

### Cell senescence assay

Primary HUVECs purchased from China center for type culture collection were used as recipient cells in cell senescence assay. The cells were incubated in endothelial cell medium (ScienceCell, #1001) supplemented with 5% fetal bovine serum (FBS; ScienceCell, #0025), 1% endothelial cell growth supplement (ECGS; ScienceCell, #1052) and 1% penicillin-streptomycin antibiotic. Senescence β-galactosidase (SA-β-gal) activity was determined using senescence β-galactosidase staining kit (Beyotime Biotechnology, C0602). Briefly, HUVECs at passage 3 were exposed to H_2_O_2_ (50 μM) for 2 h to induce senescence [35] and then incubated with 0 μg/mL or 20 *μ*g/mL ADSC-NVs for 24 h. After fixed with fixative solution for 20 minutes, HUVECs were incubated with staining solution (10 μL solution A + 10 *μ*L solution B + 930 *μ*L solution C + 50 *μ*L X-gal) at 37 °C overnight. Stained cells were visualized under a standard bright field microscope, and a total of 5–6 microscope fields were randomly selected to determine senescent HUVECs.

To further investigated the effect of ADSC-NVs on cell senescence, HUVECs at passage 3 cultured with complete medium were incubated with or without 20 μg/mL ADSC-NVs for 24 h. Senescent HUVECs were stained using SA-β-gal staining kit and the percentage of stained cells was determined of each group.

### Fat graft model

All experiments were carried out following guidelines by the Ethics Committee of Huazhong University of Science and Technology. The fat graft model was established in accordance with a previously described protocol [2, 36]. Briefly, the Coleman technique [37] was utilized to harvest the adipose tissue for transplantation from the abdomen of patients. Six-week-old male BALB/c nude mice (Shulaibao Biotech) were randomly assigned to five groups (*n* = 5 mice per group): control group (administered with phosphate-buffered saline), 5 μg/mL ADSC-NVs group, 20 μg/mL ADSC-NVs group, 80 *μ*g/mL ADSC-NVs group and 20 *μ*g/mL ADSC-EVs group. The total volume of each graft was 550 μL. The control group was injected with a mixture of 500 *μ*L of human fat and 50 *μ*L of PBS, whereas the other groups were injected with 500 *μ*L of human fat, followed by 50 *μ*L of PBS containing 5, 20, and 80 *μ*g/mL ADSC-NVs or 20 *μ*g/mL ADSC-EVs, respectively. The injection was performed using 18-G needles in the dorsal region of nude mice (two sites of each mouse) and then a 6–0 nylon suture was used to sew up the incisions. Mice were sacrificed at 12 weeks post-transplantation, followed by harvesting and weighing the grafts.

### Histological analysis

Harvested tissues were fixed with paraformaldehyde for 24 h and then embedded in paraffin. Next, paraffin-embedded tissues were sectioned, and stained with hematoxylin and eosin (H&E). Slides used for immunofluorescent staining were incubated with primary perilipin (Cell Signaling Technology, 9349S) and CD31 (Rolex-Bio, RBGB11068–2) antibodies, and subsequently incubated with secondary antibody horseradish peroxidase (HRP)-conjugated goat anti-rabbit IgG antibody (Servicebio, G1213). Finally, nuclei were counterstained with DAPI stain, and then perilipin-positive adipocytes and CD31-positive blood vessels were quantified using ImageJ software.

### Statistical analyses

All statistical analyses were performed using GraphPad Prism 8. Error bars in graphical data represent mean ± SEM. Statistical differences between groups were determined using either unpaired or two-tailed *t*-test, whereas differences among multiple groups were compared using one-way analysis of variance (ANOVA). *P* values < 0.05 were considered statistically significant.

## Results

### Generation and characterization of ADSC-NVs

**Fig. 2:**
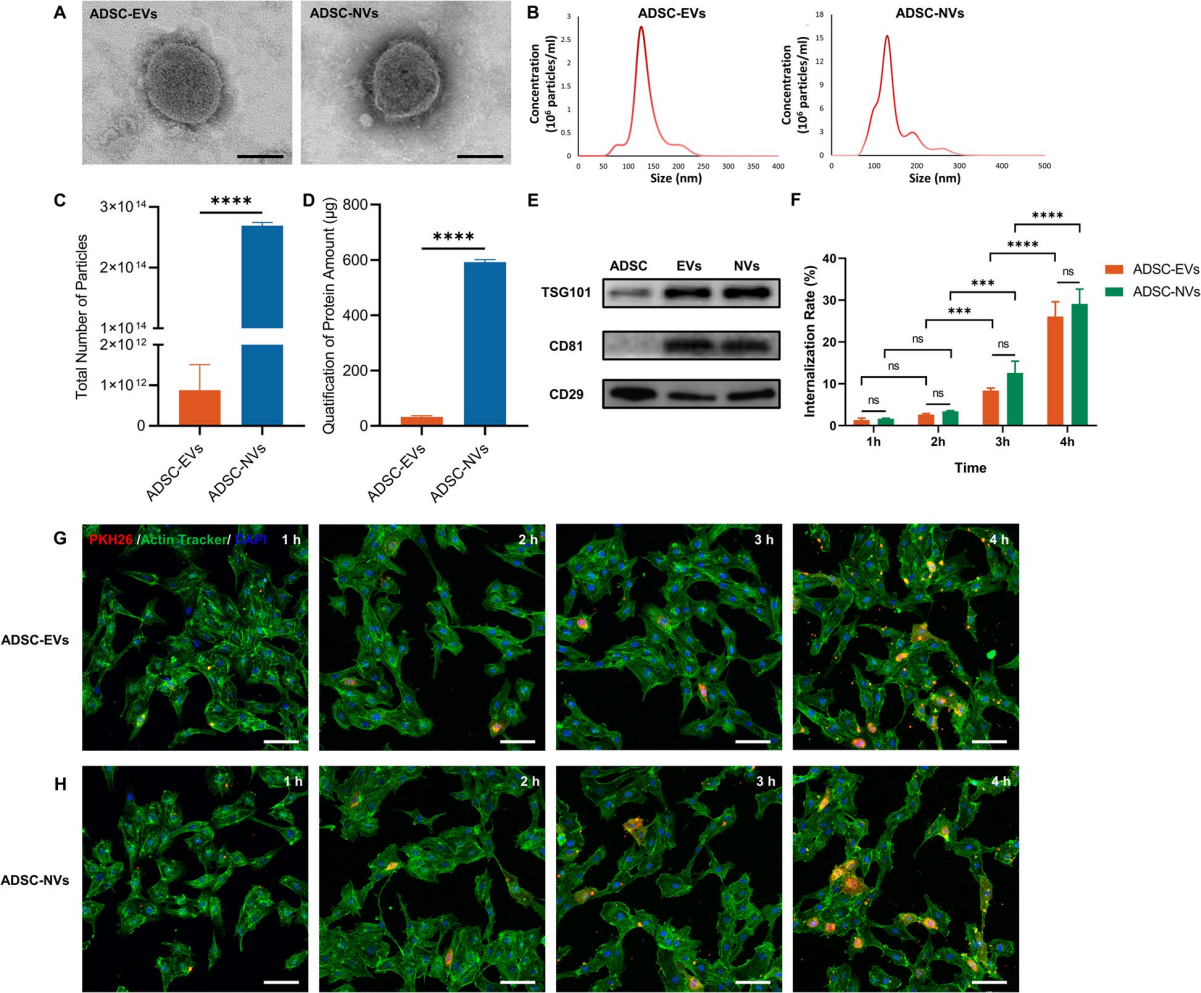
Generation and characterization of ADSC-EVs and ADSC-NVs. **A** TEM images of ADSC-EVs and ADSC-NVs. Scale bar: 100 nm. **B** NTA analysis of ADSC-EVs and ADSC-NVs. The peak diameter of both ranged between 100 and 150 nm. **C, D** The yields of EVs and NVs from equivalent ADSCs were examined by particle number (**C**) and the protein amount (**D**) (*n* = 3 per group). **E** Protein expression of TSG101, CD81, and CD29 was assessed by western blot analysis for ADSC-EVs (EVs), ADSC-NVs (NVs), and ADSC. **F-H** The internalization of PKH26-labeled ADSC-EVs and ADSC-NVs in equivalent amount particles by HUVECs was evaluated at 1, 2, 3 and 4 h respectively. The internalization rate (**F**) was measured by calculating area ratio between ADSC-EVs/ ADSC-NVs and HUVECs (*n* = 3 per group). **G, H** Representative images of internalization of ADSC-EVs or ADSC-NVs at 1, 2, 3, 4 h. Scale bar: 100 μm. Red: ADSC-EVs/NVs, Green: HUVECs, blue: Nucleus of HUVECs. ns, no significant difference, ***p < 0.001, ****p < 0.0001

ADSC-EVs were isolated by differential ultracentrifugation from an EVs-free culture medium for hADSCs. ADSC-NVs were prepared using a sequential extrusion method that involved extruding hADSCs through polycarbonate membrane micro-filters with progressively decreasing apertures (10 μm, 5 *μ*m, and 1 *μ*m), followed by iodixanol density gradient ultracentrifugation. The morphology, size distribution, yield, and protein contents of purified ADSC-EVs and ADSC-NVs were assessed. Transmission electron microscope (TEM) images revealed that ADSC-NVs consisted of 100–150 nm spherical vesicles enclosed by a bilayer lipid membrane, which was similar to the known morphology of ADSC-EVs (Fig. 2A). The nanoparticle tracking analysis (NTA) of ADSC-EVs and ADSC-NVs confirmed a similar size distribution with a central diameter ranging between 100 and 150 nm (ADSC-EVs: 141.4 ± 6.3 nm, ADSC-NVs: 142.2 ± 5.4 nm, Fig. 2B). The production efficiency of purified ADSC-NVs and ADSC-EVs from 3 × 10^7^ ADSCs was determined based on particle number and protein levels. When the total number of particles was compared, there were approximately 140 times more ADSC-NVs than ADSC-EVs (Fig. 2C). In addition, ADSC-NVs exhibited a ~ 20-fold increase in protein content compared to ADSC-EVs (Fig. 2D), indicating an increase in yield with the extrusion strategy. As recommended by *Minimal information for studies of extracellular vesicles 2018 (MISEV2018)* [38], typical extracellular vesicle proteins such as tumor susceptibility gene 101 (TSG101), a specific EV marker relevant to intracellular trafficking, and the endosomal pathway [39], and the transmembrane protein CD81, were detected in both ADSC-NVs and ADSC-EVs (Fig. 2E). A representative surface marker of ADSCs, CD29, was detected in ADSCs, ADSC-EVs, and ADSC-NVs (Fig. 2E). Extracellular vesicles can be internalized and their contents transferred to recipient cells [40, 41]. HUVECs were treated with PKH26-labeled ADSC-NVs or ADSC-EVs in equivalent number of particles for 1, 2, 3 and 4 h to confirm the internalization of ADSC-NVs and ADSC-EVs. Confocal images revealed that both ADSC-EVs and ADSC-NVs were initially internalized within an hour and gradually accumulated in treated cells as treatment time extended (Fig. 2G, H). The internalization rate of ADSC-EVs and ADSC-NVs at different points was further measured by calculating area ratio between ADSC-EVs/ ADSC-NVs and HUVECs. After 4 h of incubation, the internalization efficiencies of ADSC-EVs and ADSC-NVs were similar (26.07 ± 2.042% and 29.10 ± 2.040% respectively, Fig. 2F). These findings demonstrated that extrusion is an effective strategy for the large-scale production of ADSC-nanovesicles. Furthermore, ADSC-NVs shared the same properties and biological internalization as ADSC-EVs.

### Promoted angiogenesis mediated by ADSC-NVs in vitro

**Fig. 3:**
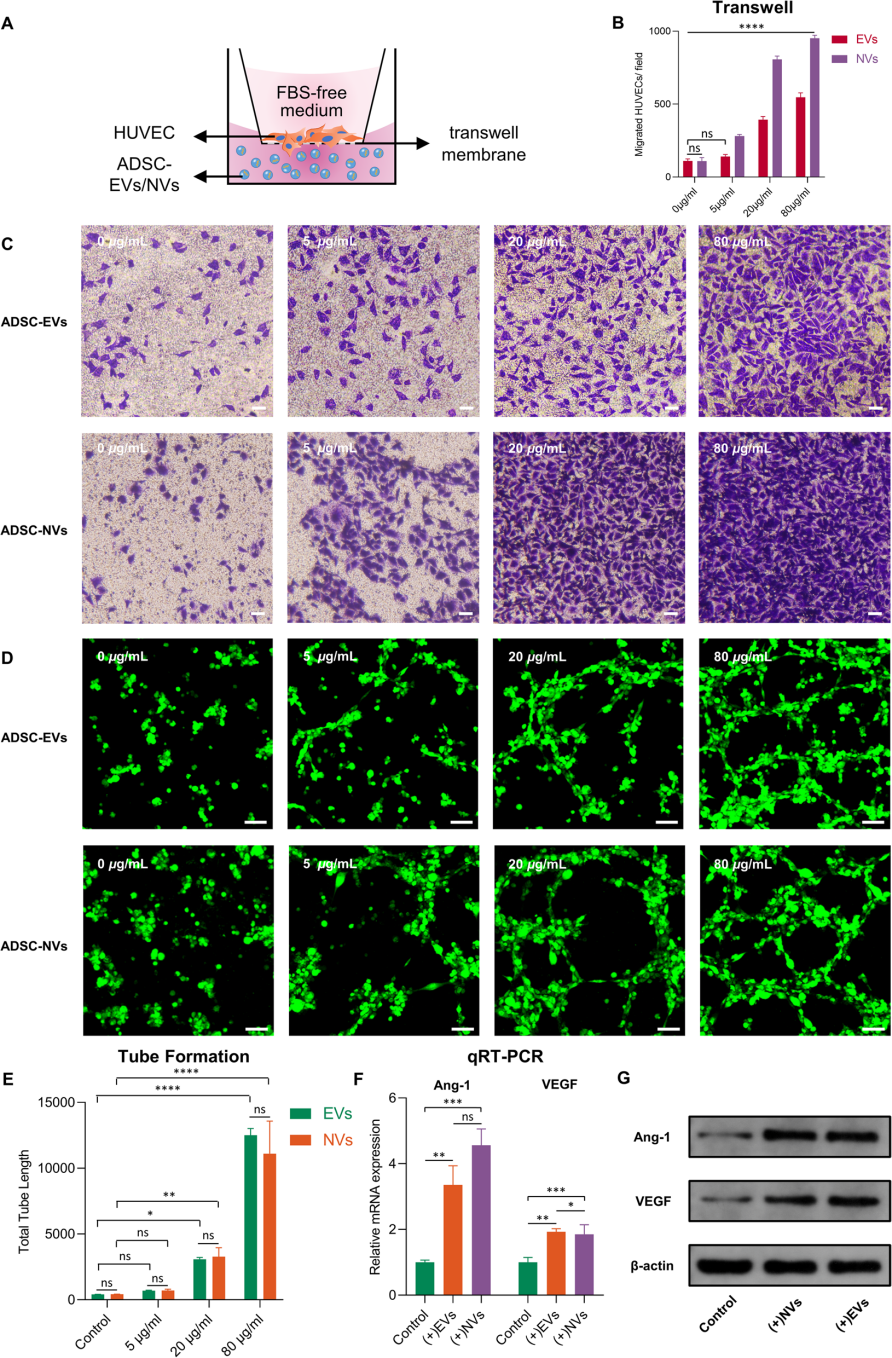
Stimulated angiogenesis mediated by ADSC-NVs in vitro. The dose-effect of ADSC-NVs and ADSC-EVs on cell migration (**C**) and tube formation (**D**) abilities of HUVECs was confirmed. Considering the possible side effects and enormous economic cost of high concentrations [28], different doses of ADSC-EVs and ADSC-NVs (ranging from 5 to 80 μg/mL) were assessed. Transwell assay was conducted following the shown schematic diagram (**A**). Briefly, HUVECs on the upper side of the transwell membrane were treated with different dose of ADSC-NVs or ADSC-EVs (0, 5, 20 and 80 μg/mL) in the lower side for 24 h. The cells migrated to the bottom of the membrane were fixed with 4% paraformaldehyde and stained with crystal violet. **C** Representative images of transwell assay with ADSC-EVs or ADSC-NVs treatment. Scale bar: 10 μm. **B** Quantitative analysis of the migrated cells (*n* = 4 per group). **D** Formation of tube-like structures by HUVECs-GFP after incubation with different doses of ADSC-EVs or ADSC-NVs (0, 5, 20, and 80 μg/mL) for 6 h. Scale bar: 100 *μ*m. **E** Quantitative analysis of tube-like structures formation (n = 3 per group). **F** mRNA expression of angiopoietin-1 (*Ang-1*) and vascular endothelial growth factor (*VEGF*) in HUVECs treated with ADSC-NVs or ADSC-EVs was confirmed using qRT-PCR (n = 3 per group). **G** Expression levels of Ang-1 and VEGF proteins in HUVECs after ADSC-NVs or ADSC-EVs stimulation. ns, no significant difference, *p < 0.05, **p < 0.01, ***p < 0.001, ****p < 0.0001

To investigate the angiogenic potency of ADSC-NVs and ADSC-EVs, different doses of ADSC-NVs and ADSC-EVs ranging from 5 to 80 μg/mL were administered to the same initial number of HUVECs, taking into consideration the potential side effects and enormous economic costs of high concentration [28]. A transwell assay was used to determine the dose-effect of ADSC-NVs and ADSC-EVs on the migratory capacity of HUVECs. HUVECs were loaded onto the upper side of the transwell membrane and after 24 h, the cells migrated to the bottom of the membrane. Cells were fixed, stained, and counted (Fig. 3A). The highest migration rate was observed in samples containing 80 μg/mL of ADSC-NVs (Fig. 3B, C). The migratory rates of the other ADSC-NVs treated groups were in descending order from 80 *μ*g/mL, 20 *μ*g/mL, 5 *μ*g/mL to 0 *μ*g/mL group. HUVECs treated with ADSC-EVs exhibited a similar dose-dependent pro-migratory effect (Fig. 3B, C). In addition, HUVECs form tube-like structures after treatment with ADSC-NVs and ADSC-EVs (Fig. 3D). Analysis of the length of the tube-like structures revealed that ADSC-NVs and ADSC-EVs stimulated tube formation activity of HUVECs in a dose-dependent manner, with 80 *μ*g/mL being the most effective (Fig. 3E). The anti-apoptosis effect of ADSC-EVs and ADSC-NVs on HUVECs was assessed using a flow cytometric assay with Annexin V-FITC / PI double staining. With ADSC-EVs/ ADSC-NVs treatment, both the early apoptosis (Annexin V+/PI-) and apoptotic (Annexin V+/PI+) rates of HUVECs were decreased (Fig. S1A). These findings demonstrated that similar to ADSC-EVs, ADSC-NVs were capable of eliciting a variety of angiogenic and anti-apoptotic behaviors in vitro. In addition, the pro-angiogenic effect of ADSC-NVs is dose-dependent.

To investigate the pro-angiogenesis and anti-apoptotic effects of ADSC-NVs, mRNA, and protein associated with angiogenesis (Ang-1 and VEGF) and apoptosis (anti-apoptotic factor Bcl-2 and pro-apoptosis factor Bax) were evaluated. HUVECs were treated for 24 h with ADSC-NVs or ADSC-EVs (20 μg/mL). Consistent with previous research, ADSC-NVs and ADSC-EVs treatment significantly increased Ang-1, VEGF, and Bcl-2 mRNA and protein levels, and decreased Bax levels (Fig. 3F, G, Fig. S1B-D). Collectively, these findings demonstrated that ADSC-NVs have pro-angiogenic and anti-apoptotic properties.

### Analysis of the microRNA expression profile in ADSC-NVs and ADSC-EVs

**Fig. 4:**
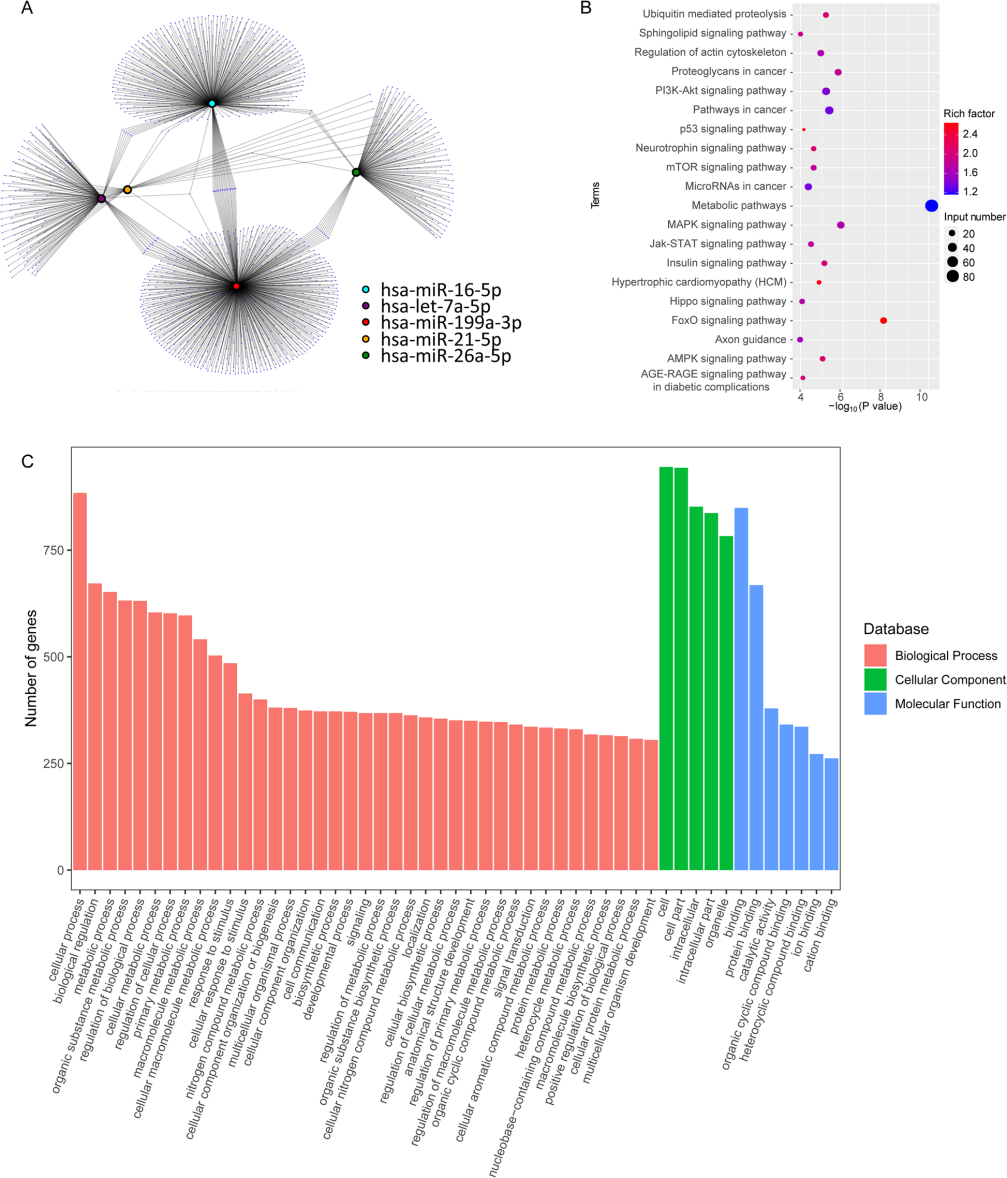
Target analysis of the top 50 highly expressed miRNAs in ADSC-NVs. **A** Cytoscape network of the interaction of the top five miRNAs abundant in ADSC-NVs and their predicted target genes. **B** KEGG pathway map of the predicted target genes of the top five miRNAs enriched in ADSC-NVs. **C** GO terms of the top five miRNAs of ADSC-NVs in biological process, cellular component, and molecular functions

**Fig. 5:**
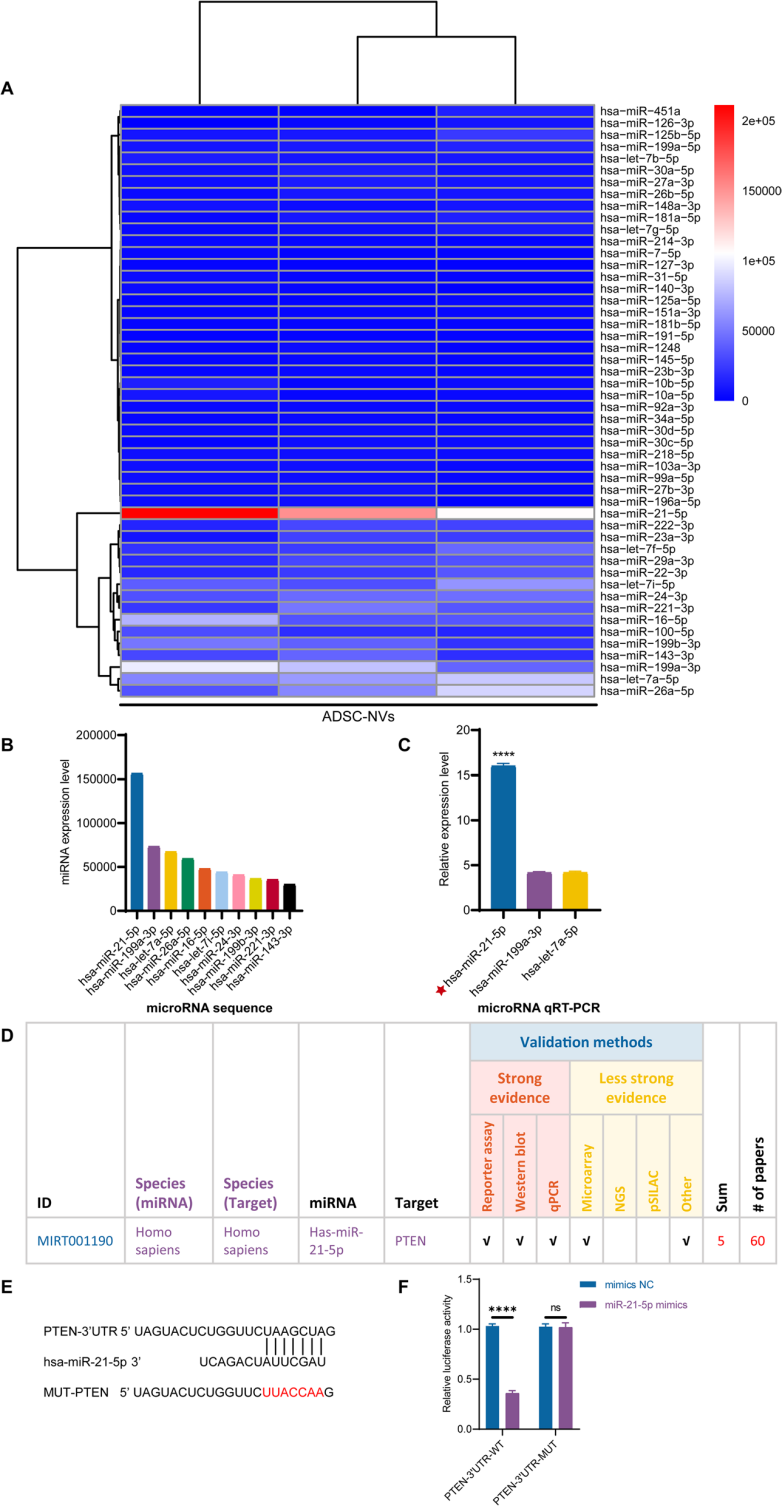
miRNA sequencing of ADSC-NVs contents and potential targets of miR-21-5p. **A** Highly expressed microRNAs of ADSC-NVs were identified by miRNA sequencing. **B** The top 10 most-enriched miRNAs in ADSC-NVs. **C** qRT-PCR analysis determined the expression level of the top three most-enriched miRNAs in ADSC-NVs (*n* = 3 per group). Results showed that miR-21-5p was the highest expressed miRNA in ADSC-NVs. Given that accumulation of miR-21 was detected in ultracentrifuged cell culture supernatant pellets and was not found in low speed centrifuged (2000 x g) pellets in a previous study [43], the high expression of miR-21-5p may be attributed to the varied molecular weight of different miRNAs. **D** miRTarBase analysis of the target genes of miR-21-5p. **E** Sequence of miR-21-5p and its predicted binding site in the 3’UTR of *PTEN* mRNA, and the mutant PTEN 3’UTR (MUT-PTEN). **F** The luciferase activity was detected in HEK-293 cells transfected with miR-21-5p mimics or control miRNA. Luciferase reporters expressing wild-type or mutant PTEN 3’UTR were used (*n* = 3 per group). ns, no significant difference, ****p < 0.0001

Recently, microRNAs were defined as the most abundant cargo RNA species in extracellular vesicles [42]. Therefore, miRNA sequencing was used to determine the global miRNA expression profile of ADSC-NVs and ADSC-EVs. As demonstrated in Fig. S2A, hierarchical clustering analysis revealed differentially expressed miRNAs in ADSC-NVs and ADSC-EVs. A Venn diagram (Fig. S2B) showed 210 miRNA species shared by ADSC-EVs and ADSC-NVs, as well as 215 up-regulated miRNAs and 123 down-regulated miRNAs in ADSC-NVs. Significantly upregulated miRNAs (fold change ≤0.5 and ≥ 2 and *P*-value ≤0.05) in ADSC-NVs (red points) and ADSC-EVs (green points) were illustrated in a volcano plot (Fig. S2C). The top 50 highly expressed miRNAs in ADSC-NVs and ADSC-EVs were respectively depicted in Fig. 5A and Fig. S3A. To further explore the functions of differentially expressed miRNAs, Cytoscape, Kyoto Encyclopedia of Genes and Genomes (KEGG), and Gene Ontology (GO) was performed. The top five miRNAs abundant in ADSC-NVs or ADSC-EVs and their predicted target genes were visualized on the Cytoscape network (Fig. 4A and Fig. S3B). The biological processes, cellular components, molecular function, and signaling pathways regulated by the top 5 miRNAs in ADSC-NVs and ADSC-EVs were identified using KEGG and GO analysis (Fig. 4B, C and Fig. S3C, D). The results showed that miRNAs in ADSC-NVs and ADSC-EVs were involved in vascular remolding signaling pathways such as the “PI3K-Akt signaling pathway”, “mitogen-activated protein kinase (MAPK) signaling pathway”, “Wnt signaling pathway,” and “Jak-STAT signaling pathway” (Fig. 4B and Fig. S3C). Besides, KEGG pathway map showed that p53 signaling pathway was a potential target for the top 5 miRNAs in ADSC-NVs (Fig. 4B). As p53 was identified as a marker for cellular senescence [44], we invested the effect of ADSC-NVs on HUVECs senescence. Firstly, HUVECs at passage 3 were exposed to H_2_O_2_ (50 μM, 2 h) to induce premature senescence [35] and then incubated with 0 μg/mL or 20 *μ*g/mL ADSC-NVs for 24 h. The SA β-gal staining showed that after H_2_O_2_ exposure, SA β-gal positive cells in enlarger and flat appearance increased to 29.2 ± 2.433%. Further incubation with ADSC-NVs did not mitigate or aggravate the senescent activity of HUVECs (Fig. S4A, B). Next, HUVECs at passage 3 cultured with complete medium were incubated with or without ADSC-NVs for 24 h and stained with SA β-gal. No significant differences of percentage of senescent HUVECs were observed after the treatment of ADSC-NVs (Fig. S4C, D). The results suggest that ADSC-NVs have negligible effect on cell senescence. The top five enriched microRNAs in ADSC-NVs and ADSC-EVs were found to be involved in the GO function annotation of biological processes (e.g., cell communication, response to stimulus), cellular components (e.g., intracellular, organelle), and molecular mechanisms (e.g., protein binding, catalytic activity) (Fig. 4C and Fig. S3D). Overall, these findings demonstrate that, while microRNA expression differs between ADSC-EVs and ADSC-NVs, they may share signaling pathways and biological processes. This could explain why ADSC-NVs and ADSC-EVs promote angiogenesis.

### miR-21-5p in ADSC-NVs promote angiogenesis by inhibiting PTEN expression and activating the PI3K/Akt pathway in HUVECs

Previous evidence suggests that miRNAs in extracellular vesicles play a role in multiple physiological and pathological processes [14]. miRNA sequencing was used to investigate the underlying mechanism for the pro-angiogenic effect of ADSC-NVs. Figure 5A shows the top 50 highly expressed miRNAs that were revealed through miRNA sequencing. Figure 5B depicts the relative expression levels of the top 10 miRNAs in ADSC-NVs. Furthermore, qRT-PCR was used to confirm the expression levels of the top 3 most abundant miRNAs (miR-21-5p, miR-199a-3p, let-7a-5p). The analysis revealed that miR-21-5p was the most-enriched miRNA in ADSC-NVs (Fig. 5C). A search in the microRNA-target interactions database miRTarBase (http://mirtarbase.cuhk.edu.cn/) was performed to predict the functions of miR-21-5p. PTEN ranked high among the predicted targets of miR-21-5p (Fig. 5D) and has been associated with vascular remodeling, cell migration, and cell apoptosis [45]. To determine if miR-21-5p binds directly to the 3’UTR region of the PTEN gene, we used the luciferase reporter assay to clone the wild-type and mutant 3’UTR of PTEN. In HEK-293 cells, co-transfection of miR-21-5p mimics with the wild-type reporter plasmids resulted in a significant decrease in luciferase activity, whereas co-transfection with mutant reporter plasmids reversed the effect (Fig. 5F). In addition, co-transfection of NC mimics with wild or mutant reporter plasmids did not affect luciferase activity (Fig. 5E, F). These findings indicated that miR-21-5p in ADSC-NVs can bind to the 3’UTR region of PTEN mRNA and suppress PTEN expression.

**Fig. 6:**
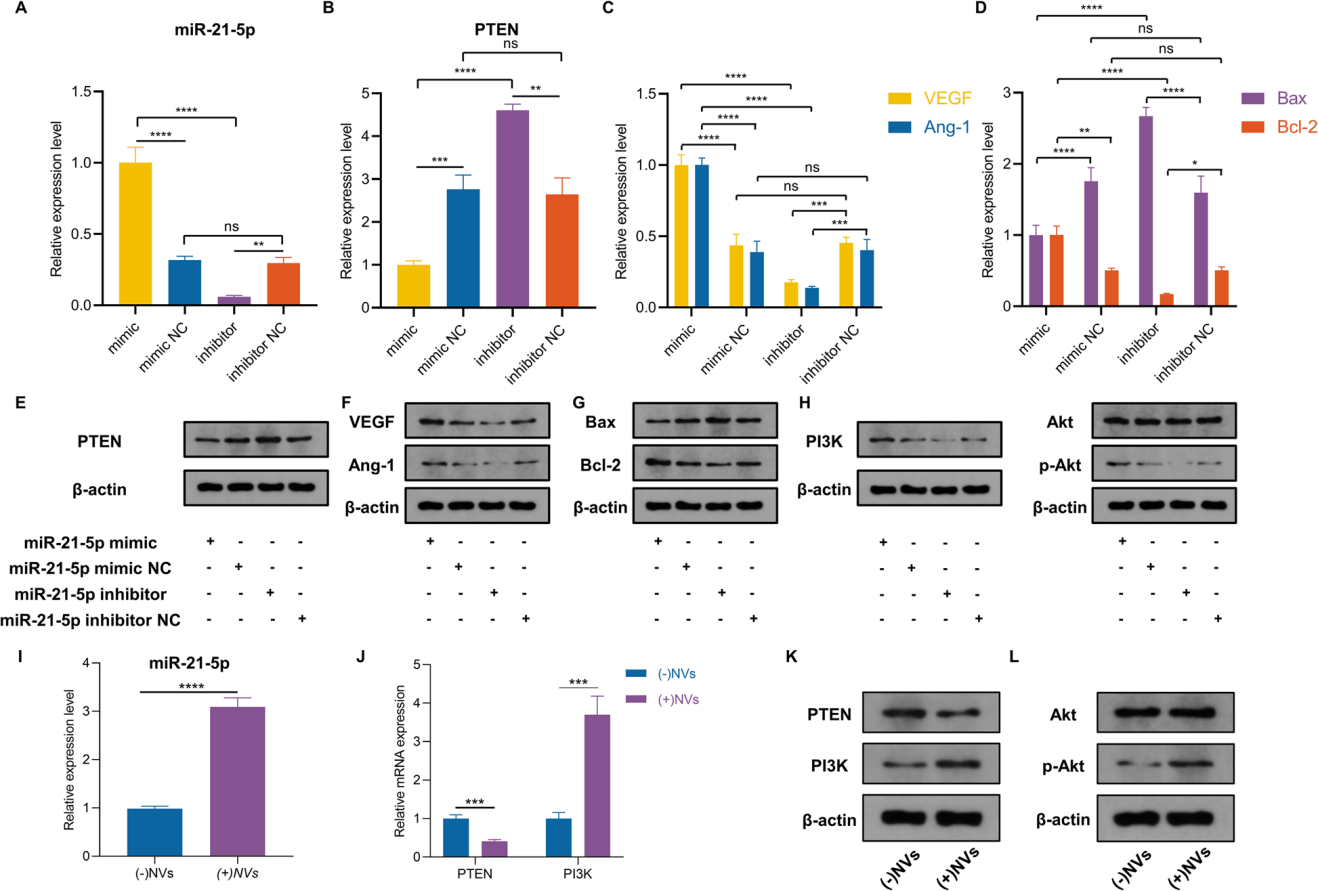
miR-21-5p abundant in ADSC-NVs inhibits PTEN and activates PI3K/AKT signaling in HUVECs. First, HUVECs were transfected with miR-21-5p mimic or inhibitor. qRT-PCR assay was then conducted to determine the relative expression level of *miR-21-5p* (**A**) and *PTEN* (**B**) in the mimic, mimic negative control (mimic NC), inhibitor, and inhibitor negative control (inhibitor NC) groups (*n* = 3 per group). qRT-PCR assay of the relative expression level of angiogenetic *VEGF* and *Ang-1* mRNA (**C**), and apoptosis associated mRNA expression of *Bax* and *Bcl-2* (**D**) in the mimic, mimic NC, inhibitor, and inhibitor NC groups (*n* = 3 per group). **E** Western blot assay for protein level of PTEN. **F-G** Western blot assay for protein level of VEGF, Ang-1, Bax, and Bcl-2. **H** Western blot assay of PI3K, Akt, and p-Akt protein level. Next, HUVECs were treated with/without ADSC-NVs for 24 h and collected for analysis. **I** qRT-PCR analysis of the relative expression level of *miR-21-5p* (n = 3 per group). **J** qRT-PCR analysis of relative expression level of *PTEN* and *PI3K* (n = 3 per group). **K** Western blot assay for protein levels of PTEN and PI3K. **L** Western blot assay for protein levels of Akt and p-Akt. ns, no significant difference, *p < 0.05, **p < 0.01, ***p < 0.001, ****p < 0.0001

To further investigate the role of miR-21-5p in pro-angiogenesis, HUVECs were transfected with miR-21-5p mimic and miR-21-5p inhibitor for 24 h. The expression level of miR-21-5p was determined by qRT-PCR. Results showed a significantly higher expression level of miR-21-5p in the mimic group compared to that of the inhibitor group (Fig. 6A). In addition, qRT-PCR and western blotting assay revealed a significant decrease in PTEN level in miR-21-5p mimic-transfected cells, which was reversed following transfection with miR-21-5p inhibitor (Fig. 6B, E). Although, miR-21-5p mimic transfection increased VEGF, Ang-1, and Bcl-2 mRNA and protein levels, miR-21-5p inhibitor transfection decreased VEGF, Ang-1, and Bcl-2 levels (Fig. 6C, D, F, G). The expression of pro-apoptotic factor Bax was inhibited in the miR-21-5p mimic group and increased in the miR-21-5p inhibitor group (Fig. 6D, G). PTEN has been identified as a PI3K/Akt signaling meditator involved in vascular remodeling [46]. Compared to the miR-21-5p inhibitor group, miR-21-5p mimic transfection increased the expression level of PI3K and p-Akt (Fig. 6H). These findings validated the potential role of miR-21-5p in promoting angiogenesis and inhibiting apoptosis by regulating the PTEN/PI3K/p-Akt signaling pathway.

Following the determination of the role of miR-21-5p in angiogenesis in vitro, we hypothesized that ADSC-NVs enhanced angiogenesis by activating the miR-21-5p/PTEN/PI3K/p-Akt pathway. When HUVECs were treated with ADSC-NVs for 24 h, there was a significant increase in miR-21-5p levels compared to non-treated cells (Fig. 6I). PTEN and PI3K mRNA and protein levels were significantly decreased and increased, respectively, in ADSC-NVs treated HUVECs (Fig. 6J, K). Western blotting analysis revealed that ADSC-NVs-treatment resulted in p-Akt accumulation (Fig. 6L). After incubation with ADSC-NVs, HUVECs exhibited higher expression of Ang-1 and VEGF, as confirmed by qRT-PCR and western blotting assay (Fig. 3F-H). These findings show that high levels of miR-21-5p enriched in ADSC-NVs promote vascularization in HUVECs by activating PI3K/p-Akt signaling.

### ADSC-NVs promote vascularized fat regeneration in the fat transplantation model by activating the PI3K/Akt pathway

**Fig. 7:**
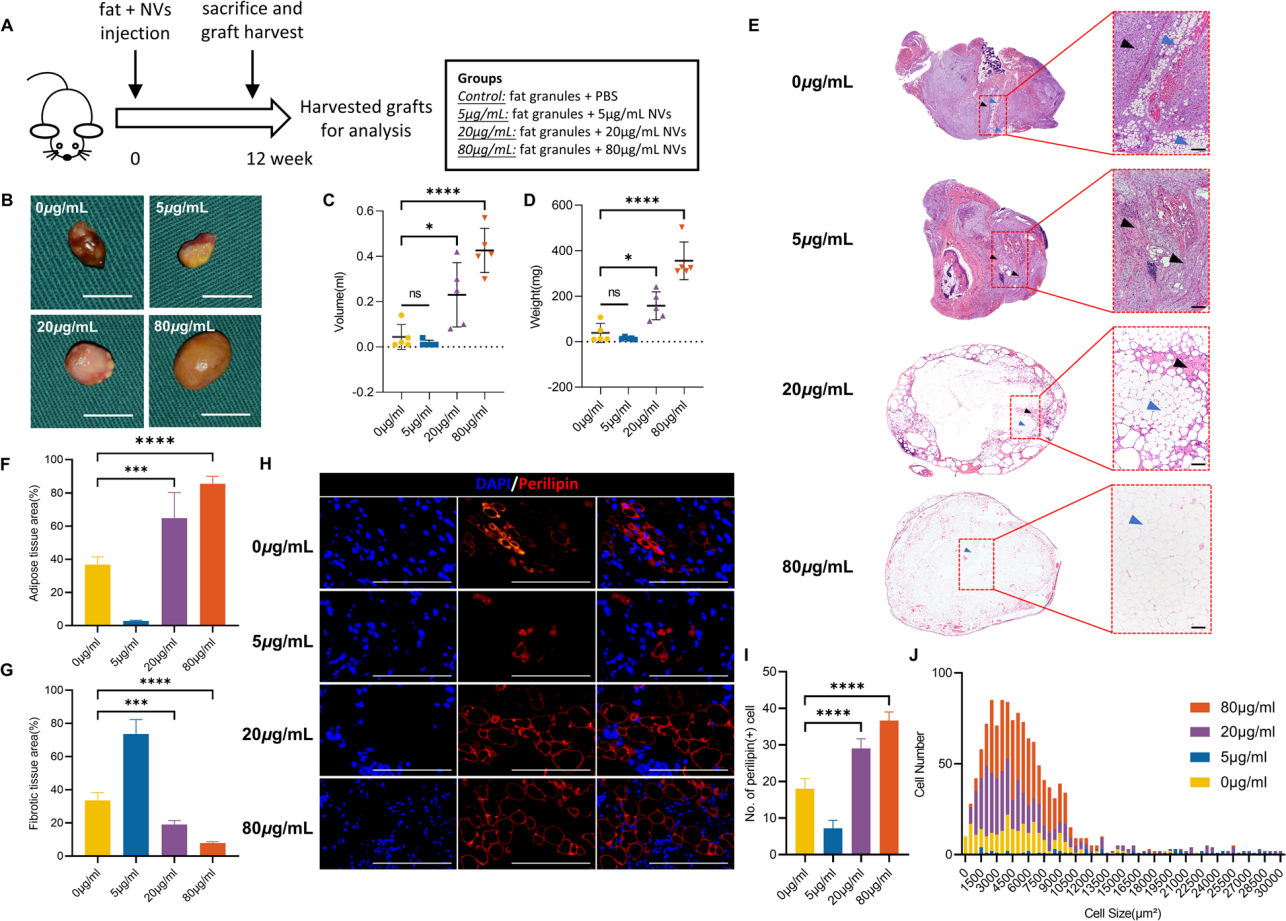
ADSC-NVs improved fat graft survival. **A** Schematic design of the animal study. Briefly, four groups of BALB/c nude mice were administered injection of a mixture of fat granules and different dose of ADSC-NVs (0, 5, 20 and 80 μg/mL). The grafts were harvested at 12 weeks post-transplantation for further analysis. **B** Macroscopic images of the fat grafts. Scale bar: 1 cm. Quantitative analysis of grafts shows higher volume (**C**) and weight (**D**) in the 20 *μ*g/mL and 80 *μ*g/mL groups (*n* = 5 per group). **E** The overall view of H&E staining. Black arrowheads, fibrosis; blue arrowheads, adipose. Scale bar: 100 μm. Quantitative analysis of H&E stained images shows that the adipose tissue area (**F**) of the 80 *μ*g/mL group was the highest, followed by the 20 *μ*g/mL group, control group, and finally the 5 *μ*g/mL group (*n* = 5 per group). The proportion of fibrotic tissue area (**G**) showed contrasting results (*n* = 4 per group). (**H**) Immunofluorescent staining for perilipin of harvested grafts to determine survival of adipocytes. Scale bar: 100 *μ*m. **I** The number of perilipin (+) adipocytes were higher in high-dose ADSC-NVs treatment groups (80 *μ*g/mL and 20 *μ*g/mL) than in control and 5 *μ*g/mL groups (*n* = 5 per group). **J** Analysis of cell size and distribution confirms adipocyte size and amount in four groups increased in order from 5 *μ*g/mL group and control group to 20 *μ*g/mL and 80 *μ*g/mL groups. ns: no significant difference, *p < 0.05, ***p < 0.001, ****p < 0.0001

**Fig. 8:**
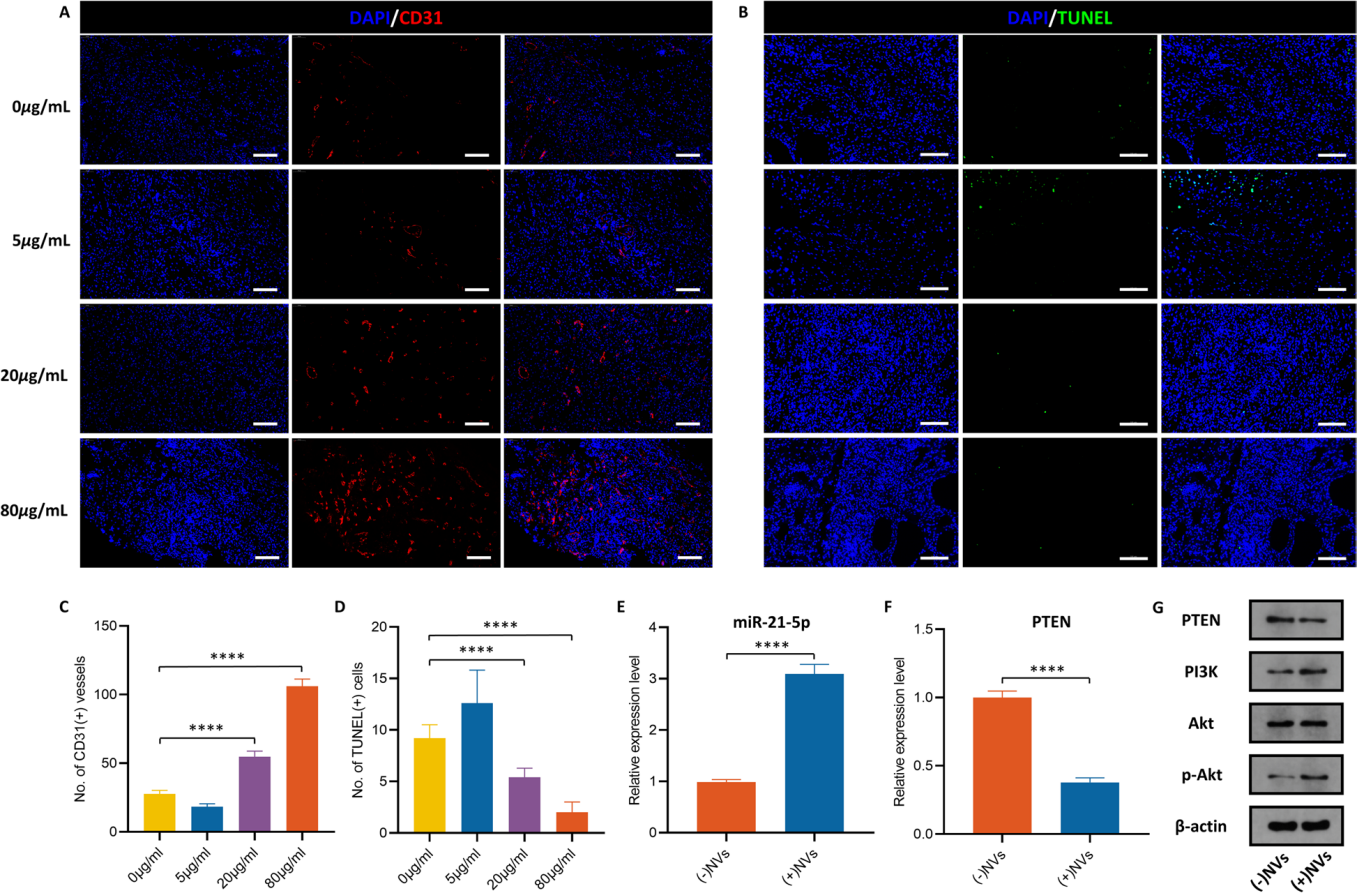
Stimulated angiogenesis mediated by ADSC-NVs in vivo. Immunofluorescent staining for CD31 (**A**) and TUNEL (**B**) of the explanted specimens to identify angiogenesis and anti-apoptosis effects, respectively. Scale bar: 50 *μ*m. **C** CD31 (+) vessel structure was the highest in 80 *μ*g/mL and 20 *μ*g/mL groups than in the control and 5 *μ*g/mL groups (*n* = 3 per group). **D** Measurement of TUNEL (+) cells indicated higher anti-apoptosis effect in the 80 *μ*g/mL and 20 *μ*g/mL groups than in the control and 5 *μ*g/mL groups (*n* = 5 per group). qRT-PCR analysis of the relative expression levels of *miR-21-5p* (**E**) and *PTEN* (**F**) in (−)NVs (control) and (+)NVs (NV-treated) groups (*n* = 3 per group). **G** Protein levels of PTEN, PI3K, Akt, and p-Akt of grafts with or without ADSC-NVs treatment. ****p < 0.0001

Autologous fat transplantation is widely applied in soft-tissue augmentation. According to previous research, sufficient blood supply during the early stages of fat grafting determines the fate of transplanted fat [2]. Animal models were implanted with fat with varying doses of ADSC-NVs (0, 5, 20, 80 μg/mL) to investigate the role of ADSC-NVs in fat regeneration in vivo. Adipose tissue remodeling was reported to significantly occur within the first 2–3 months [47]; therefore, grafts were harvested after 12 weeks for analysis (Fig. 7A). Figure 7B illustrates representative images from each group. All grafts were encased in a thin fibrous membrane. Grafts from groups with high concentrations of ADSC-NVs (20, 80 μg/mL) had significantly higher volumes (0 *μ*g/mL group, 0.044 ± 0.055 mL; 5 *μ*g/mL group, 0.016 ± 0.0134 mL; 20 *μ*g/mL group, 0.230 ± 0.142 mL; 80 *μ*g/mL group, 0.426 ± 0.098 mL) and wet weights (0 *μ*g/mL group, 38.52 ± 42.14 mg; 5 *μ*g/mL group, 14.58 ± 5.07 mg; 20 *μ*g/mL group, 158.12 ± 61.49 mg; 80 *μ*g/mL group, 344.64 ± 82.97 mg) compared to those of the control group and 5 μg/mL group (Fig. 7C, D). To investigate the efficacy of ADSC-NVs and ADSC-EVs in fat regeneration, fat graft with 20 μg/mL ADSC-EVs was carried out. Quantitative analysis of grafts showed no significant difference of volume (ADSC-NVs group, 0.230 ± 0.142 mL; ADSC-EVs group, 0.1980 ± 0.1031 mL) and weight (ADSC-NVs group, 158.12 ± 61.49 mg; ADSC-EVs, 171.2 ± 88.88 mg) between ADSC-NVs group and ADSC-EVs group in the same concentration (20 *μ*g/mL) (Fig. S5A-C). Histological analysis was further performed in grafts with ADSC-NVs. H&E staining analysis revealed excessive fibrosis in fat grafts from the control and 5 μg/mL group, accompanied by cell infiltration of various cells (Fig. 7E). In contrast, fat grafts in the 20 and 80 *μ*g/mL groups showed significant adipose tissue regeneration and less fibrous tissue. Magnified images revealed more adipocytes in the 20 and 80 *μ*g/mL groups with regular shapes than in the control and 5 *μ*g/mL groups. Black arrows represent fibrosis and blue arrows represent adipose. In addition, the quantitative analysis demonstrated more adipose tissue and fewer fibrotic tissue region in the 20 and 80 μg/mL groups compared to the control and 5 *μ*g/mL groups (Fig. 7F, G). A “necrotic zone” was observed in the control and 5 μg/mL groups. This zone represents the region of dead cells after transplantation that has less regeneration and can be filled with fibrous tissue formation [36]. Perilipin is a protein that coats lipid droplets in adipocytes and can be used to identify living adipocytes [48]. Perilipin immunofluorescent staining was used to explore the structure of *via*ble adipocytes (Fig. 7H). Fat grafts from the 20 and 80 *μ*g/mL groups had more perilipin-positive crown-like structures than those from the control and 5 *μ*g/mL groups. This finding was confirmed by quantitative analysis (Fig. 7I). The morphology of adipocytes in H&E staining and perilipin staining was used to further characterize viable adipocytes. Histological analysis may not reveal nuclei of adipocytes due to the large size of adipocytes (50 ~ 150 μm diameter). Therefore, to avoid misclassifying large cavities as adipocytes, round lipid droplets within 30,000 *μ*m^2^ were labeled as healthy adipocytes. Cell size distribution analysis revealed that adipocytes in the 20 and 80 μg/mL groups were more in number and homogeneous in shape, with sizes ranging from 2000 to 6000 *μ*m^2^ (Fig. 7J). In summary, these findings demonstrate that ADSC-NVs promote adipocyte survival in vivo. To further explore the biological role of ADSC-NVs in adipose retention, we performed CD31 immunofluorescent staining of the explanted specimens to detect vascular endothelial cells. The density of CD31(+) cells surrounding the vascular fractions was higher in groups with better fat survival (20 and 80 *μ*g/mL group) compared to the control and 5 *μ*g/mL groups (Fig. 8A, C). The results of the anti-apoptosis effect using the TUNEL assay showed fewer TUNEL (+) apoptotic cells in 20 and 80 *μ*g/mL groups compared to the control and 5 *μ*g/mL groups (Fig. 8B, D). The expression level of miR-21-5p in the harvested grafts was determined to further explore the pro-angiogenesis role of miR-21-5p in fat regeneration. qRT-PCR analysis revealed that the expression of miR-21-5p was increased in the ADSC-NVs treated group (Fig. 8E). Consistent with the in vitro results, the ADSC-NVs treated group exhibited higher levels of PI3K and p-Akt, whereas PTEN was significantly suppressed when compared to the control group (Fig. 8F, G). These findings imply that ADSC-NVs have a role in promoting fat tissue regeneration via the pro-angiogenesis activity of miR-21-5p.

## Discussion

The current gold standard for MSC-EV purification is ultracentrifugation [49]; it is a time-consuming procedure requiring a mass of parent cells. Moreover, MSCs produce extracellular vesicles in small quantities, approximately ~ 5 μg/mL per day [19]. To achieve therapeutic outcomes, typically a dose of 10^9^ ~ 10^11^ EVs is needed in the treatment in mice as reported [20], which may require time-consuming conditioned media collection and cell expansion. The production yield of ADSC-NVs from 3 × 10^7^ ADSCs in our study is 2.7 × 10^14^ ± 5.03 × 10^12^ (Fig. 2C). Based on our experience, 20 mL tissue from healthy female donors via lipo-aspiration could harvest ~ 3.3 × 10^7^ ADSCs at passage 3. It has been reported that MSCs could maintain the proliferation profile, cell cycle distribution and MSC surface markers expression until passage 10 [50]. In addition, the genomic stability is somehow guaranteed in MSCs during in vitro long-term culture [51]. Thus, the characteristics and activity of ADSCs used to generate NVs in this study are relatively stable. Therefore, we could infer that this extrusion strategy to generate ADSC-NVs with high yield production could effectively avoid cell senescence and its convenient feature makes it potential for clinical application. The difference in biogenesis between engineered nanovesicles and extracellular vesicles could explain the improved yield efficiency of ADSC-NVs: EVs are known to capture particular compositions based on a selective cargo-sorting theory [21], whereas NVs form by self-assembly of lipid bilayer pieces from fragmentized cells into vesicles. The self-assembly process may trigger a non-selective encapsulation of any contents in the surrounding medium. In addition, only a portion of the cell contents may be secreted in EVs, but NVs theoretically may contain whole cell contents.

The biological function of NVs has been reported to be similar to that of extracellular vesicles. Evidence suggests that BMSC-NVs, like BMSC-EVs, may enhance osteogenesis and stimulate skeletal regeneration [19]. ADSC-EVs can sustain the viability and proliferation of endothelial cells [52]. Thus, we hypothesized that ADSC-NVs, like ADSC-EVs, might stimulate angiogenesis by inducing migration and tube formation in related cell types such as HUVECs. In support of this hypothesis, the present study confirmed the overlapping pro-angiogenesis effect of ADSC-NVs and ADSC-EVs, which positively correlated with the concentration of ADSC-NVs and ADSC-EVs. HUVECs exhibited comparable angiogenic capacity when treated with the same concentration of ADSC-NVs or ADSC-EVs. Considering the higher yield efficiency of ADSC-NVs, it may be possible to stimulate angiogenesis more effectively in practice. When combined with the molecular biology findings, this study confirms the role of ADSC-NVs in promoting angiogenesis via a synergetic combination of increased migration, tube formation, and decreased apoptosis.

The variation in miRNA profiles between ADSC-EVs and ADSC-NVs can be attributed to their different biogenesis, as previously stated. Extracellular vesicles are formed by the endosomal system or are shed from the plasma membrane [21], whereas the formation of engineered nanovesicles might be a non-selective encapsulation of its parent cells. KEGG assay of their target genes revealed that miRNA species abundant in ADSC-NVs and ADSC-EVs were strongly associated with vascular remolding, despite their distinct miRNA expression profiles. Let-7 family members, in particular, are associated with the JAK-STAT signaling pathway to induce angiogenesis [53], accounting for ~ 16% and ~ 23% of the total miRNAs in ADSC-NVs and ADSC-EVs, respectively. In addition, MAPK signaling shared by ADSC-NVs and ADSC-EVs (Fig. 4B and Fig. S3C) was demonstrated to control actomyosin contractility and thus regulate angiogenesis and other biological processes [54]. Overall, while microRNA expression differs between ADSC-EVs and ADSC-NVs, they may share signaling pathways and biological processes. This could explain why ADSC-NVs and ADSC-EVs promote angiogenesis.

Previous research indicates that miRNAs in extracellular vesicles can be transferred into recipient cells, whereby they can mediate multiple signaling pathways, eventually modulating cellular functions [55]. In addition, research demonstrates that miRNAs in EVs are potential biomarkers of disease, and are implicated in the stimulation of angiogenesis, cardiac stem cell proliferation, and antioxidant effects [14]. In this view, we hypothesized that ADSC-NVs could promote angiogenesis by transferring miRNAs to recipient cells and then regulating associated gene expression and bioactivity. Overexpression of miR-21-5p was first observed in various cancers and was associated with tumor metastasis [43]. Recent evidence shows that high levels of miR-21-5p in MSC-EVs play a role in protecting the ischemic myocardium from apoptosis. However, the direct role of miR-21 in regulating angiogenesis in tissue regeneration remains elusive. To the best of our knowledge, this is the first study to examine miR-21-5p levels in ADSC-NVs. The findings of this study show that miR-21-5p is a pro-angiogenesis and anti-apoptosis agent, as transfection of miR-21-5p mimics resulted in high expression levels of VEGF, Ang-1, and Bcl-2 proteins. Accumulation of miR-21-5p in ADSC-NVs may be attributed to the ultracentrifugation purification method. A previous study explored the expression level of miR-21-5p in 2000×g pellet and 100,000×g EVs [43]. In contrast to the 100,000×g pellet, accumulation of miR-21 was not detected in the 2000×g pellet. PI3K/Akt signaling is critical for cell survival, microvascular permeability, and angiogenesis [46]. PTEN negatively regulates the PI3K/Akt signaling pathway by removing the 3′ phosphate of PIP3. Studies have shown that PTEN is a promising target for inducing apoptosis and combating vascular remolding [56]. To validate the pro-angiogenesis and anti-apoptosis effect of miR-21-5p, we hypothesized that miR-21-5p would activate PI3K/Akt signaling by inhibiting PTEN. To confirm this hypothesis, molecular analysis revealed that inhibiting miR-21-5p induced the expression of PTEN, ultimately suppressing PI3K/Akt signaling. In addition, ADSC-NVs-treatment significantly decreased PTEN and caused PI3K and p-Akt accumulation. Therefore, we proposed a molecular determinant in ADSC-NVs, including, but not limited to miR-21-5p, that could promote angiogenesis in vitro by blocking PTEN and activating the PI3K/Akt signaling pathway.

Adipose tissue has a dense capillary network that connects each adipocyte. Ischemia and hypoxia of adipose tissue following transplantation may result in a cascade of complications such as inflammation, cyst formation, and necrosis [36]. As a result, vascularization determines the fate of transplanted fat and adipogenesis. This study revealed that ADSC-NVs significantly increased graft vascularization, resulting in a high-fat tissue retention rate. In addition, as demonstrated by the TUNEL assay, the anti-apoptosis effect of ADSC-NVs may facilitate the survival of adipocytes and ADSCs through the remodeling process.

Intriguingly, grafts in the 5 μg/mL group had little effect on vascularization and fat regeneration. A previous study investigated the anti-angiogenesis effect of extracellular vesicles from different cell types via CD36-mediated signaling [57]. This could explain why repressive vascularization occurs under high ADSC-EVs concentrations [2]. These angiogenesis-inhibiting factors may be lost during the formation of ADSC-NVs. Therefore, for a dose of ADSC-NVs ~ 5 *μ*g/mL, side effects may outweigh the benefit of pro-vascularization to adipose regeneration, and this effect may be reversed by higher concentrations of ADSC-NVs. Further research is warranted to explore the contents of ADSC-EVs and ADSC-NVs to better understand their functions.

Numerous studies have verified the therapeutic effect of MSC-EVs in tissue regeneration [58]. To investigate the efficacy of ADSC-EVs and ADSC-NVs in fat tissue regeneration, we analyzed the fat grafts treated with ADSC-EVs or ADSC-NVs in the same concentration (20 *μ*g/mL). This study revealed that both ADSC-NVs and ADSC-EVs could increase the fat tissue retention rate. Consistent with our study in vitro, ADSC-NVs and ADSC-EVs had comparable efficacy in fat tissue regeneration in vivo. As previously reported, the promotion of MSC-EVs in tissue repair might be due to its ability to regulate neovascularization and alleviate inflammatory response [2].

A cell-free therapeutic technique was developed following the discovery that the conditioned medium obtained from gene-edited cells has cytoprotective properties [59]. MSC-EVs-based intervention for regenerative therapy is currently popular due to its high transfection efficiency in a wide variety of cells, long-term storage capacity, and ready-to-use superiority. As a consistent progression of cell-free therapy, MSC-NVs with expanding production can improve treatment of diseases that require large amounts of MSC-EVs to function effectively, for example, diabetes, myocardial ischemic, cerebral infarction and so on.

## Conclusion

Although MSC-EVs are gaining traction in regenerative medicine, insufficient yield continues to impede their clinical application. To shatter this hindrance, we generated ADSC-NVs on a mass scale by s sequentially extruding ADSCs through micro-filters with diminishing pore size. In addition, sufficient vascularization is a prerequisite for tissue regeneration. MSC-EVs derived from different sources exhibited various biological features. In this study, ADSC-NVs inherited the angiogenesis-induction property from ADSC-EVs, which was regulated by miR-21-5p enriched in ADSC-NVs. miR-21-5p exhibited its activity through suppression of PTEN thus activating PI3K/Akt signaling. In addition, the administration of ADSC-NVs in fat transplantation models significantly increased vascularization and adipose regeneration. In the future, ADSC-NVs may be used as an alternative to ADSC-EVs for cell-free treatment in regenerative medicine.

## Supplementary Information

**Additional file 1: Fig. S1.** Anti-apoptosis mediated by ADSC-NVs in vitro. (A) Viability of ADSC-NVs or ADSC-EVs treated HUVECs was measured by Annexin-V–FITC/PI staining. We defined Annexin V+/PI- as early apoptotic stage, Annexin V+/PI+ as apoptotic cells, and Annexin V−/PI+ as dead cells. Total apoptotic rate was the sum of early apoptotic rate and apoptotic rate. (B, C) mRNA expressions of B-cell lymphoma-2 (Bcl-2) (B) and BCL2-associated X (Bax) (C) in HUVECs treated with ADSC-NVs or ADSC-EVs were analyzed using qRT-PCR (n = 3 per group). (D) Protein levels of Bcl-2 and Bax in HUVECs after ADSC-NVs or ADSC-EVs stimulation. *p < 0.05, **p < 0.01, ***p < 0.001, ****p < 0.0001.

**Additional file 2: Fig. S2.** Differential microRNA expression profile of ADSC-NVs and ADSC-EVs. (A) Hierarchical clustering assay of differentially expressed miRNAs between ADSC-NVs and ADSC-EVs. (B) Venn diagram showing 210 shared miRNA species between ADSC-EVs and ADSC-NVs, 215 upregulated miRNAs and 123 downregulated miRNAs in ADSC-NVs. (C) Volcano plot of miRNAs in ADSC-NVs and ADSC-EVs. The red points indicate significantly upregulated miRNAs (fold change ≤0.5 and ≥ 2 and *P* value ≤0.05) in ADSC-NVs, whereas green points indicate upregulated miRNAs in ADSC-EVs.

**Additional file 3: Fig. S3.** Target analysis of the top 50 highly expressed miRNAs in ADSC-EVs. (A) Heatmap of the top 50 highly expressed miRNAs in ADSC-EVs. (B) Cytoscape network of the interaction of the top five miRNAs abundant in ADSC-EVs and their predicted target genes (blue). (C) KEGG pathway map of the predicted target genes of the top five miRNAs enriched in ADSC-EVs. (D) GO terms of top five miRNAs of ADSC-EVs in biological process, cellular component, and molecular functions.

**Additional file 4: Fig. S4.** Negligible effect of ADSC-NVs on cell senescence in vitro. HUVECs were exposed to H_2_O_2_ (50 μM, 2 h) to induce senescence and then incubated with 0 μg/mL or 20 *μ*g/mL ADSC-NVs for 24 h. (A) Representative images of SA β-gal staining of HUVECs. Scale bar: 100 μm. (B) Quantitation of SA β-gal-positive HUVECs (n = 3 per group). Besides, HUVECs cultured in complete medium were incubated with or without 20 *μ*g/mL ADSC-NVs for 24 h and stained with SA β-gal. (C) Representative images of SA β-gal staining of HUVECs. Scale bar: 100 *μ*m. (D) Quantitation of SA β-gal-positive HUVECs (n = 3 per group). ns, no significant difference, **p < 0.01, ***p < 0.001.

**Additional file 5: Fig. S5.** Fat graft with ADSC-EVs. BALB/c nude mice were administered injection of a mixture of fat granules and ADSC-EVs (20 *μ*g/mL). The grafts were harvested at 12 weeks post-transplantation for further analysis. (A) Macroscopic images of the fat grafts. Scale bar: 1 cm. Quantitative analysis of grafts shows no significant difference of volume (B) and weight (C) between ADSC-NVs (20 *μ*g/mL) group and ADSC-EVs (20 *μ*g/mL) group (n = 5 per group). ns, no significant difference.

## Data Availability

Not applicable.

## Abbreviations

MSC-EVs: Mesenchymal stem cell-derived extracellular vesicles
ADSC-NVs: Adipose-derived stem cell engineered nanovesicles
ADSC-EVs: Adipose-derived stem cell-derived extracellular vesicles
hADSCs: Human ADSCs
MSCs: Mesenchymal stem cells
3D: Three-dimensional
NVs: Nanovesicles
ES cells: Embryonic stem cells
PBS: Phosphate-buffered saline
FBS: Fetal bovine serum
EVs: Extracellular vesicles
TEM: Transmission electron microscope
HUVEC: Human umbilical vein endothelial cell
PI: Propidium iodide
GO: Gene ontology
KEGG: Kyoto Encyclopedia of Genes and Genomes
qRT-PCR: Quantitative reverse transcription-polymerase chain reaction
SA β-gal: Senescence β-galactosidase
NC: Negative control
H&E: Hematoxylin and eosin
NTA: Nanoparticle tracking analysis
TSG101: Tumor susceptibility gene 101
Ang-1: Angiopoietin-1
VEGF: Vascular endothelial growth factor
MAPK: Mitogen-activated protein kinase
