# Supplementary material for: High yield engineered nanovesicles from ADSC with enriched miR-21-5p promote angiogenesis in adipose tissue regeneration
Source: Biomater Res. 2022 Dec 17;26:83. doi: 10.1186/s40824-022-00325-y (PMC9758932; doi:10.1186/s40824-022-00325-y)
Supplement: Supplementary file 1 — Additional file 1: Fig. S1. Anti-apoptosis mediated by ADSC-NVs in vitro. (A) Viability of ADSC-NVs or ADSC-EVs treated HUVECs was measured by Annexin-V–FITC/PI staining. We defined Annexin V+/PI- as early apoptotic stage, Annexin V+/PI+ as apoptotic cells, and Annexin V−/PI+ as dead cells. Total apoptotic rate was the sum of early apoptotic rate and apoptotic rate. (B, C) mRNA expressions of B-cell lymphoma-2 (Bcl-2) (B) and BCL2-associated X (Bax) (C) in HUVECs treated with ADSC-NVs or ADSC-EVs were analyzed using qRT-PCR (n = 3 per group). (D) Protein levels of Bcl-2 and Bax in HUVECs after ADSC-NVs or ADSC-EVs stimulation. *p < 0.05, **p < 0.01, ***p < 0.001, ****p < 0.0001. [file 40824_2022_325_MOESM1_ESM.pdf]

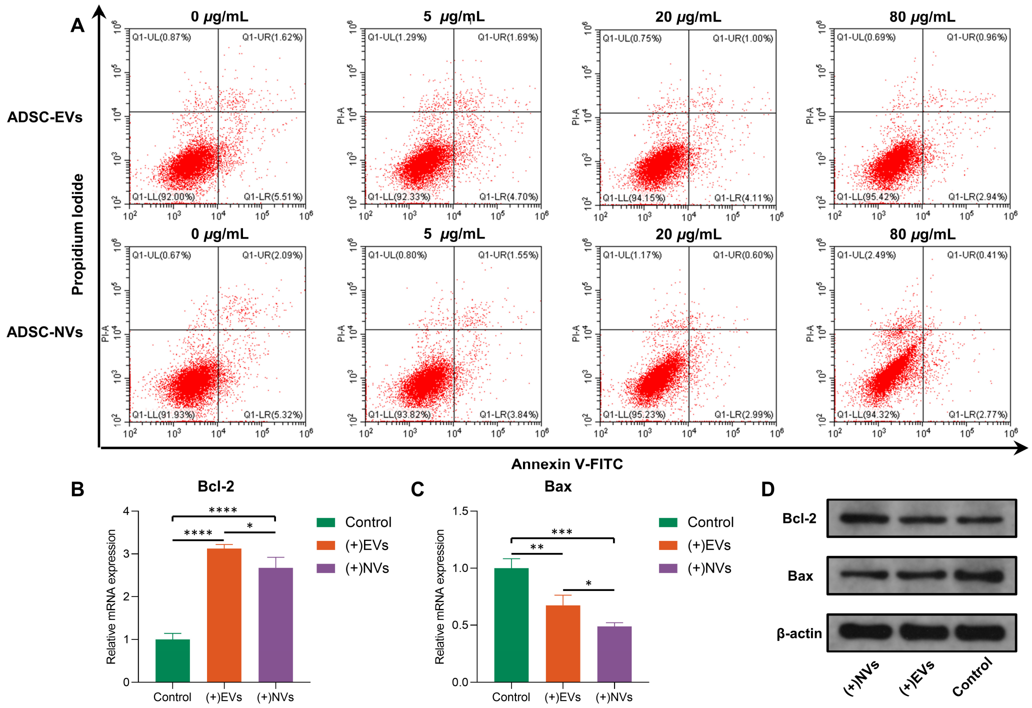

Figure S1: Anti-apoptosis mediated by ADSC-NVs *in vitro*. (A) Viability of ADSC-NVs or ADSC-EVs treated HUVECs was measured by Annexin-V–FITC/PI staining. We defined Annexin V+/PI- as early apoptotic stage, Annexin V+/PI+ as apoptotic cells, and Annexin V-/PI+ as dead cells. Total apoptotic rate was the sum of early apoptotic rate and apoptotic rate. (B, C) mRNA expressions of B-cell lymphoma-2 (Bcl-2) (B) and BCL2-associated X (Bax) (C) in HUVECs treated with ADSC-NVs or ADSC-EVs were analyzed using qRT-PCR (n = 3 per group). (D) Protein levels of Bcl-2 and Bax in HUVECs after ADSC-NVs or ADSC-EVs stimulation. *p < 0.05, **p < 0.01, ***p < 0.001, ****p<0.0001.
