# Supplementary material for: High yield engineered nanovesicles from ADSC with enriched miR-21-5p promote angiogenesis in adipose tissue regeneration
Source: Biomater Res. 2022 Dec 17;26:83. doi: 10.1186/s40824-022-00325-y (PMC9758932; doi:10.1186/s40824-022-00325-y)
Supplement: Supplementary file 2 — Additional file 2: Fig. S2. Differential microRNA expression profile of ADSC-NVs and ADSC-EVs. (A) Hierarchical clustering assay of differentially expressed miRNAs between ADSC-NVs and ADSC-EVs. (B) Venn diagram showing 210 shared miRNA species between ADSC-EVs and ADSC-NVs, 215 upregulated miRNAs and 123 downregulated miRNAs in ADSC-NVs. (C) Volcano plot of miRNAs in ADSC-NVs and ADSC-EVs. The red points indicate significantly upregulated miRNAs (fold change ≤0.5 and ≥ 2 and P value ≤0.05) in ADSC-NVs, whereas green points indicate upregulated miRNAs in ADSC-EVs. [file 40824_2022_325_MOESM2_ESM.pdf]

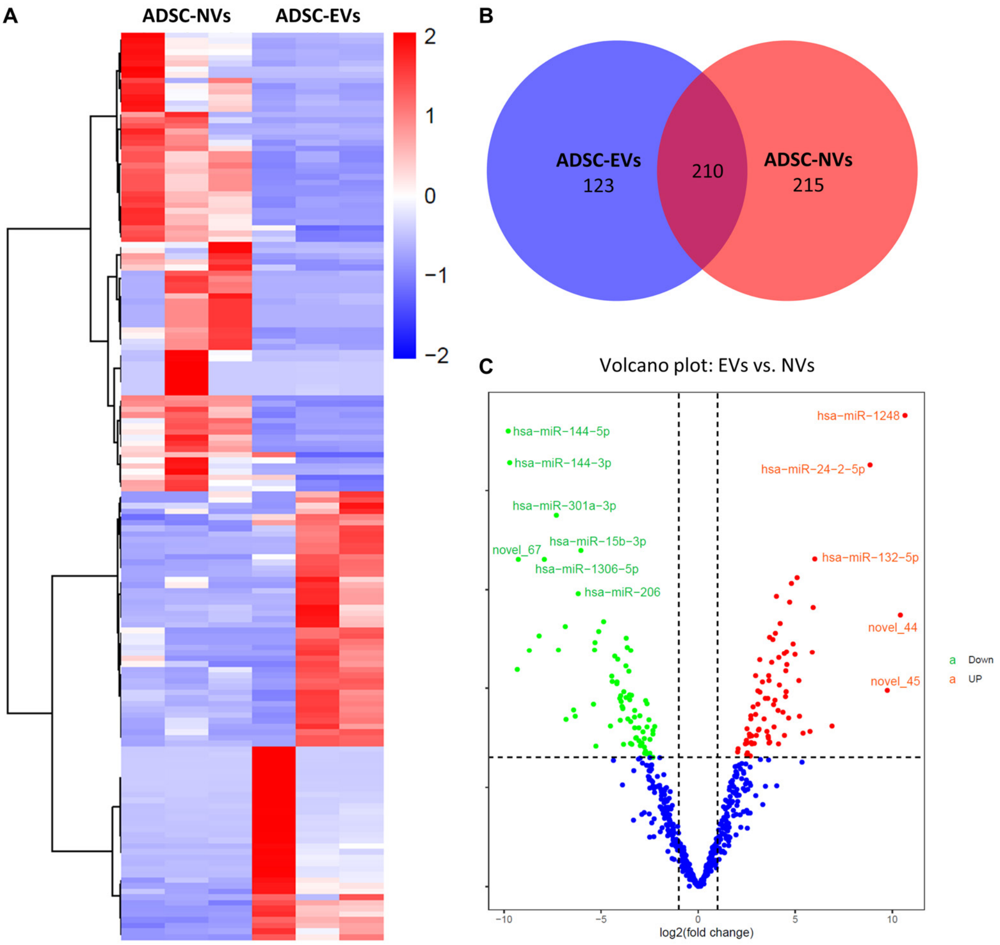

Figure S2: Differential microRNA expression profile of ADSC-NVs and ADSC-EVs. (A) Hierarchical clustering assay of differentially expressed miRNAs between ADSC-NVs and ADSC-EVs. (B) Venn diagram showing 210 shared miRNA species between ADSC-EVs and ADSC-NVs, 215 upregulated miRNAs and 123 downregulated miRNAs in ADSC-NVs. (C) Volcano plot of miRNAs in ADSC-NVs and ADSC-EVs. The red points indicate significantly upregulated miRNAs (fold change ⩽ 0.5 and ⩾ 2 and P value ⩽ 0.05) in ADSC-NVs, whereas green points indicate upregulated miRNAs in ADSC-EVs.
