# Supplementary material for: High yield engineered nanovesicles from ADSC with enriched miR-21-5p promote angiogenesis in adipose tissue regeneration
Source: Biomater Res. 2022 Dec 17;26:83. doi: 10.1186/s40824-022-00325-y (PMC9758932; doi:10.1186/s40824-022-00325-y)
Supplement: Supplementary file 3 — Additional file 3: Fig. S3. Target analysis of the top 50 highly expressed miRNAs in ADSC-EVs. (A) Heatmap of the top 50 highly expressed miRNAs in ADSC-EVs. (B) Cytoscape network of the interaction of the top five miRNAs abundant in ADSC-EVs and their predicted target genes (blue). (C) KEGG pathway map of the predicted target genes of the top five miRNAs enriched in ADSC-EVs. (D) GO terms of top five miRNAs of ADSC-EVs in biological process, cellular component, and molecular functions. [file 40824_2022_325_MOESM3_ESM.pdf]

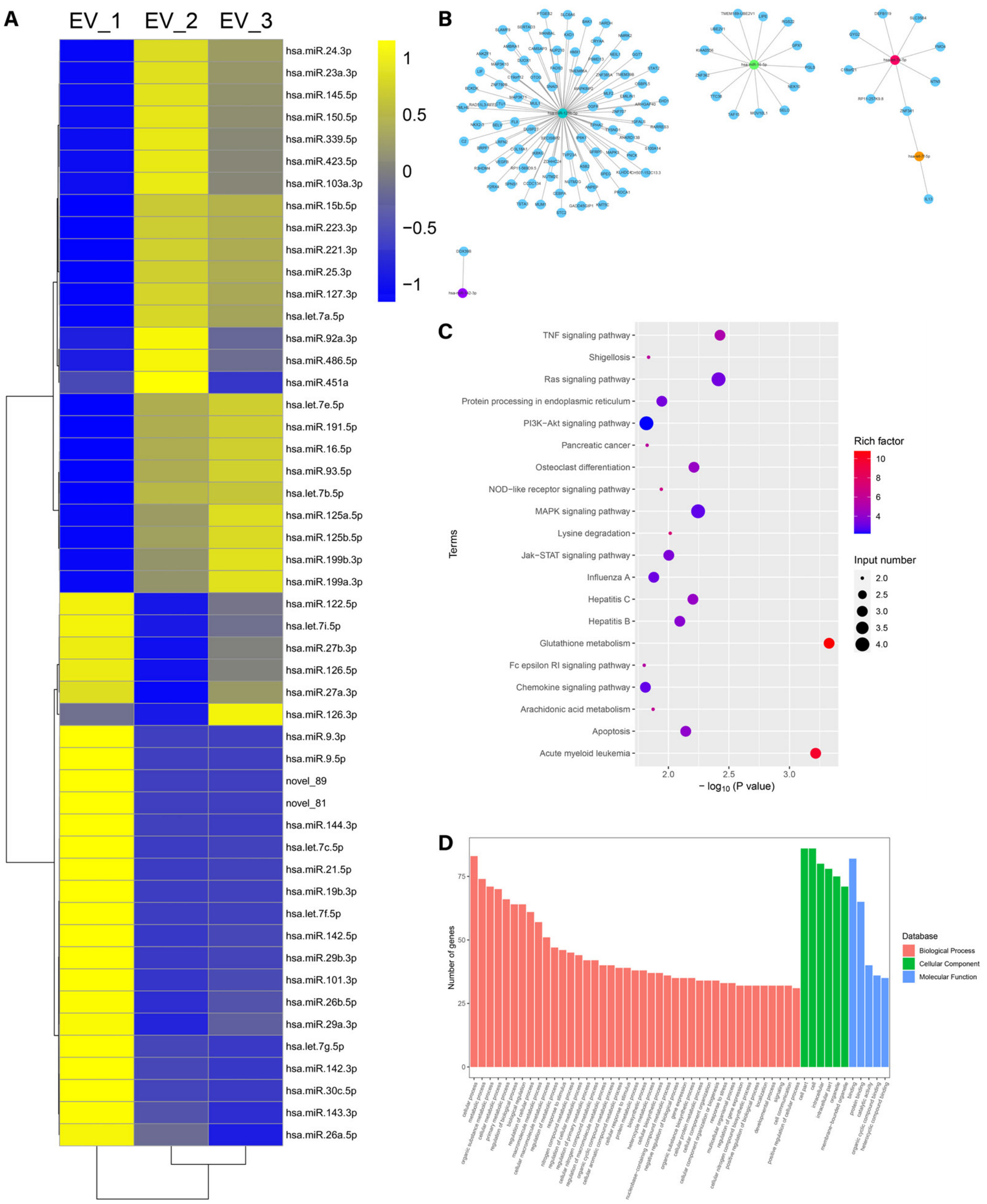

Figure S3: Target analysis of the top 50 highly expressed miRNAs in ADSC-EVs. (A) Heatmap of the top 50 highly expressed miRNAs in ADSC-EVs. (B) Cytoscape network of the interaction of the top five miRNAs abundant in ADSC-EVs and their predicted target genes (blue). (C) KEGG pathway map of the predicted target genes of the top five miRNAs enriched in ADSC-EVs. (D) GO

terms of top five miRNAs of ADSC-EVs in biological process, cellular component, and molecular functions.
