# Supplementary material for: High yield engineered nanovesicles from ADSC with enriched miR-21-5p promote angiogenesis in adipose tissue regeneration
Source: Biomater Res. 2022 Dec 17;26:83. doi: 10.1186/s40824-022-00325-y (PMC9758932; doi:10.1186/s40824-022-00325-y)
Supplement: Supplementary file 4 — Additional file 4: Fig. S4. Negligible effect of ADSC-NVs on cell senescence in vitro. HUVECs were exposed to H2O2 (50 μM, 2 h) to induce senescence and then incubated with 0 μg/mL or 20 μg/mL ADSC-NVs for 24 h. (A) Representative images of SA β-gal staining of HUVECs. Scale bar: 100 μm. (B) Quantitation of SA β-gal-positive HUVECs (n = 3 per group). Besides, HUVECs cultured in complete medium were incubated with or without 20 μg/mL ADSC-NVs for 24 h and stained with SA β-gal. (C) Representative images of SA β-gal staining of HUVECs. Scale bar: 100 μm. (D) Quantitation of SA β-gal-positive HUVECs (n = 3 per group). ns, no significant difference, **p < 0.01, ***p < 0.001. [file 40824_2022_325_MOESM4_ESM.pdf]

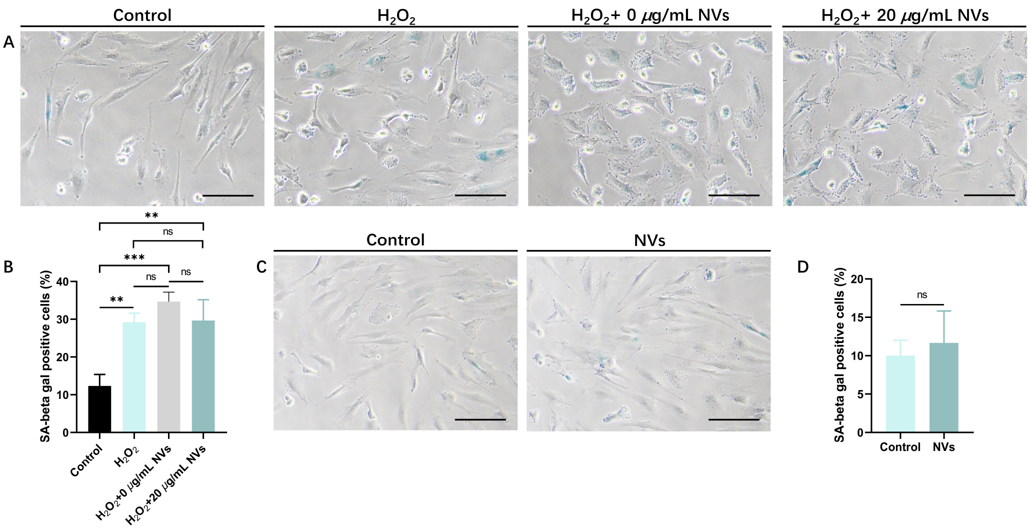

Figure S4: Negligible effect of ADSC-NVs on cell senescence *in vitro*. HUVECs were exposed to H_2O_2 (50 μM, 2h) to induce senescence and then incubated with 0 μg/mL or 20μg/mL ADSC-NVs for 24 h. (A) Representative images of SA β-gal staining of HUVECs. Scale bar: 100 μm. (B) Quantitation of SA β-gal-positive HUVECs (n = 3 per group). Besides, HUVECs cultured in complete medium were incubated with or without 20μg/mL ADSC-NVs for 24 h and stained with SA β-gal. (C) Representative images of SA β-gal staining of HUVECs. Scale bar: 100 μm. (D) Quantitation of SA β-gal-positive HUVECs (n = 3 per group). ns, no significant difference, **p < 0.01, ***p < 0.001.
