# Supplementary material for: High yield engineered nanovesicles from ADSC with enriched miR-21-5p promote angiogenesis in adipose tissue regeneration
Source: Biomater Res. 2022 Dec 17;26:83. doi: 10.1186/s40824-022-00325-y (PMC9758932; doi:10.1186/s40824-022-00325-y)
Supplement: Supplementary file 5 — Additional file 5: Fig. S5. Fat graft with ADSC-EVs. BALB/c nude mice were administered injection of a mixture of fat granules and ADSC-EVs (20 μg/mL). The grafts were harvested at 12 weeks post-transplantation for further analysis. (A) Macroscopic images of the fat grafts. Scale bar: 1 cm. Quantitative analysis of grafts shows no significant difference of volume (B) and weight (C) between ADSC-NVs (20 μg/mL) group and ADSC-EVs (20 μg/mL) group (n = 5 per group). ns, no significant difference. [file 40824_2022_325_MOESM5_ESM.pdf]

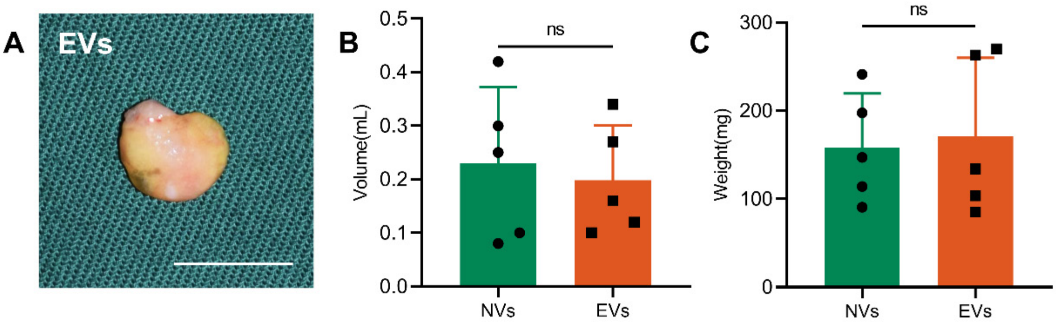

Figure S5: Fat graft with ADSC-EVs. BALB/c nude mice were administered injection of a mixture of fat granules and ADSC-EVs (20 *μ*g/mL). The grafts were harvested at 12 weeks post-transplantation for further analysis. (A) Macroscopic images of the fat grafts. Scale bar: 1 cm. Quantitative analysis of grafts shows no significant difference of volume (B) and weight (C) between ADSC-NVs (20 *μ*g/mL) group and ADSC-EVs (20 *μ*g/mL) group (n = 5 per group). ns, no significant difference.
